# Hyaluronic acid-ibuprofen conjugation: a novel ototherapeutic approach protecting inner ear cells from inflammation-mediated damage

**DOI:** 10.3389/fphar.2024.1355283

**Published:** 2024-02-15

**Authors:** Bhaskar Birru, Joachim G. S. Veit, Elizabeth M. Arrigali, Jack Van Tine, Emma Barrett-Catton, Zachary Tonnerre, Philippe Diaz, Monica A. Serban

**Affiliations:** ^1^ Department of Biomedical and Pharmaceutical Sciences, University of Montana, Missoula, MT, United States; ^2^ Montana Biotechnology Center (BIOTECH), University of Montana, Missoula, MT, United States

**Keywords:** inflammation, hyaluronic acid, ibuprofen, cochlea, ototherapeutic

## Abstract

There is a substantial need of effective drugs for the treatment of hearing loss, which affects nearly 500 million individuals globally. Hearing loss can be the result of intense or prolonged noise exposure, ototoxic drugs, infections, and trauma, which trigger inflammatory signaling cascades that lead to irreversible damage to cochlear structures. To address this, we developed and characterized a series of covalent conjugates of anti-inflammatory drugs to hyaluronic acid (HA), for potential use as topical ototherapeutics. These conjugates were tested in *in vitro* assays designed to mirror physiological processes typically observed with acoustic trauma. Intense noise exposure leads to macrophage recruitment to the cochlea and subsequent inflammatory damage to sensory cells. We therefore first tested our conjugates’ ability to reduce the release of inflammatory cytokines in macrophages. This anti-inflammatory effect on macrophages also translated to increased cochlear cell viability. In our initial screening, one conjugate, ibuprofen-HA, demonstrated significantly higher anti-inflammatory potential than its counterparts. Subsequent cytokine release profiling of ibuprofen-HA further confirmed its ability to reduce a wider range of inflammatory markers, to a greater extent than its equivalent unconjugated drug. The conjugate’s potential as a topical therapeutic was then assessed in previously developed tympanic and round window membrane tissue permeation models. As expected, our data indicate that the conjugate has limited tympanic membrane model permeability; however, it readily permeated the round window membrane model and to a greater extent than the unconjugated drug. Interestingly, our data also revealed that ibuprofen-HA was well tolerated in cellular and tissue cytocompatibility assays, whereas the unconjugated drug displayed significant cytotoxicity at equivalent concentrations. Moreover, our data highlighted the importance of chemical conjugation of ibuprofen to HA; the conjugate had improved anti-inflammatory effects, significantly reduced cytotoxicity, and is more suitable for therapeutic formulation. Overall, this work suggests that ibuprofen-HA could be a promising safe and effective topical ototherapeutic for inflammation-mediated cochlear damage.

## 1 Introduction

Cochlear damage from insults such as acoustic trauma, is a multifaceted degenerative phenomenon caused by a cascade of detrimental responses such as oxidative stress, inflammation, and excitotoxicity, which ultimately result in both necrotic and apoptotic cell death (Bohne et al., 2007; Yang et al., 2016; Kalinec et al., 2017; Arrigali and Serban, 2022; Paik et al., 2022; Xu et al., 2023). Both reactive oxygen species and inflammation have been identified as major contributors to hearing loss (Li et al., 2023). Several studies have shown that noise-induced inflammatory responses in the cochlea are triggered by the recruitment of inflammatory cells such as macrophages, and the upregulation of pro-inflammatory cytokines (Frye et al., 2019; Weiwei et al., 2020; Hough et al., 2022). Macrophages have been identified in the cochlea along the lateral wall and within the spiral limbus, and when activated through a variety of stressors (Zhang et al., 2012; Fujioka et al., 2014), they migrate to the scala tympani (Frye et al., 2017; Kalinec et al., 2017). Various studies have identified some of the inflammatory cytokine and gene expression patterns in noise-induced inflammatory conditions; however, the specific mechanisms of the damage have not been fully elucidated. Recent studies have reported that excessive noise exposure activates NF-κB signaling, which then upregulates the expression of the pro-inflammatory cytokines IL-6 and TNF-α (Riva et al., 2007; Bas Infante et al., 2012; Warnecke et al., 2019). Additional studies have shown that acoustic trauma in mice stimulates TNF-α production by the macrophages, leading to hair cell (cochlear sensory cell) death and consequently, hearing loss (Dhukhwa et al., 2019). Various anti-inflammatory approaches have been investigated for the management of hearing loss; for example, anti-inflammatory steroids such as dexamethasone, metformin, and etanercept (an FDA-approved TNF-α inhibitor), have been shown to reduce inflammatory cytokines and protect against hearing loss (Zhou et al., 2013; Han et al., 2015; Dhukhwa et al., 2019; Gedik et al., 2020).

While systemic treatments against hearing loss have been extensively explored in the past, they can be problematic due to limited drug concentrations reaching the target tissue, premature metabolic deactivation, and increased risk of adverse off-target effects (Nyberg et al., 2019). To address these shortcomings, localized drug delivery to the target tissue would be expected to improve drug availability, therapeutic efficiency, and safety. However, anatomical membranes such as the tympanic membrane (TM), which separates the outer ear and the middle ear, and the round window membrane (RWM), which separates the middle ear and the inner ear, pose significant challenges to topical drug delivery.

In this study, our approach to the development of potential topical treatments was to use hyaluronic acid (HA) as a drug carrier for well-established anti-inflammatory molecules. HA is a glycosaminoglycan ubiquitous to mammalian systems, and is found in extracellular matrix, synovial fluid, vitreous humor, connective tissue, and respiratory mucosa (Kim et al., 2020). A previous study using HA as an additive for cochlear drug delivery, found improved therapeutic outcomes in patients with sensorineural hearing loss (Rogha and Kalkoo, 2017). Additionally, our group has shown that covalent conjugation of antioxidants to HA leads to enhanced protection of cochlear hair cells from oxidative stress (Arrigali and Serban, 2022).

These advantages, coupled with the inherent chemical moieties of HA which allow for convenient chemical modification and functionalization (Serban and Skardal, 2019), make it an ideal drug carrier for use in this study. Therefore, we synthesized a series of covalently attached HA-anti-inflammatory conjugates (HAC) using several anti-inflammatory drugs; hydrocortisone (HC), prednisolone (PS), and ibuprofen (IBU). These drugs were chosen due to their well-established clinical use, and the presence of a carboxylic acid moiety in their structure, which allows for subsequent conjugation via carbodiimide chemistry. As a first step in the synthesis process, HA was enriched with carboxyl functionalities to provide additional drug binding sites, while separately, a primary amine moiety was added to the anti-inflammatory molecules to allow for subsequent carbodiimide chemical conjugation. To then assess their anti-inflammatory potential, lipopolysaccharide (LPS)-stressed macrophages were treated with HACs and the resulting inflammatory cytokine release was profiled. Only one conjugate, ibuprofen-HA (I-HA), showed anti-inflammatory effects and was further explored with cochlear cells (HEI-OC1, immortalized mouse organ of Corti cell line) (Kalinec et al., 2003; Kalinec et al., 2016; Arrigali and Serban, 2022). Interestingly, our data revealed that I-HA was well tolerated by cells despite unconjugated drug showing cytotoxic effects. Additionally, I-HA was also able to reduce macrophage-mediated inflammatory cytotoxicity in HEI-OC1 cells. Finally, I-HA permeation and tissue viability studies were conducted using *in vitro* TM and RWM permeation models previously developed by our group (Veit et al., 2022a; Singh et al., 2022). These permeation studies further highlighted the potential of I-HA as a topical therapeutic for inflammation-mediated hearing loss.

## 2 Materials and methods

### 2.1 Materials

The following cell lines, cell culture reagents and assay kits have been used for this study: House Ear Institute-organ of Corti (HEI-OC1, Kalinec Lab, UCLA, Los Angeles, CA), RAW264.7 (TIB-71, ATCC, Manassas, VA), fetal bovine serum (FBS, Corning, Corning, NY), Dulbecco’s modified eagle medium (DMEM, Corning, Corning, NY), CellTiter 96 AQueous One Solution cell proliferation assay (MTS assay, Promega, Madison, WI), CyQUANT lactate dehydrogenase (LDH) cytotoxicity assay (Invitrogen, Waltham, MA), Dulbecco’s phosphate buffered-saline (DPBS, Corning, Corning, NY), trypsin-ethylenediaminetetraacetic acid (trypsin-EDTA, Corning, Corning, NY), TNF-α mouse instant ELISA kit (**#**BMS607-2INST, Invitrogen, Waltham, MA), IL-6 mouse ELISA kit (#KMC0061, Invitrogen, Waltham, MA), V-PLEX proinflammatory panel 1 mouse kit (#K15048D Meso Scale Discovery (MSD), Rockville, MD), lipopolysaccharide (LPS, *E. Coli* O111:B4, Millipore Sigma, St. Louis, MO), mouse IL-10 recombinant protein (550,070, BD Biosciences, Franklin Lakes, NJ, USA), mouse IL-12p70 recombinant protein (Thermo Scientific, Waltham, MA), mouse TNF-α recombinant protein (#RMTNFAI Invitrogen, Waltham, MA), mouse IL-6 recombinant protein (#RMIL6I Invitrogen, Waltham, MA).

The following reagents and consumables have been used for this study: HA 20 kDa (Lifecare Biomedical, Chaska, MN), 1-ethyl-3-(3-dimethylaminopropyl) carbodiimide hydrochloride (EDC, Thermo Scientific, Waltham, MA), sodium chloride (NaCl, Thermo Scientific, Waltham, MA), sodium hydroxide (NaOH, Thermo Scientific, Waltham, MA), ethanol (EtOH, VWR, Radnor, PA), iodoacetic acid (Thermo Scientific, Waltham, MA), isopropanol (VWR, Radnor, PA), #2 Whatman paper (Thermo Scientific, Waltham, MA), deuterated water (D_2_O, Thermo Scientific, Waltham, MA), dimethyl sulfoxide (DMSO, Thermo Scientific, Waltham, MA) D_6_-DMSO (Thermo Scientific, Waltham, MA), 70 mL 3,500 kDa MW cut-off (MWCO) dialysis cassettes (Thermo Scientific, Waltham, MA), t-butyl carbazate (Thermo Scientific, Waltham, MA), 4-(dimethylamino)pyridine (DMAP, TCI Chemicals, Philadelphia, PA), N,N-diisopropylethylamine (TCI Chemicals, Philadelphia, PA), sodium sulfate (TCI Chemicals, Philadelphia, PA), ammonium chloride (NH_4_CL, TCI Chemicals, Philadelphia, PA), sodium bicarbonate (NaHCO_3,_ TCI Chemicals, Philadelphia, PA), dichloromethane (DCM, Thermo Scientific, Waltham, MA), ethyl acetate (Thermo Scientific, Waltham, MA), trifluoroacetic acid (TFA, Thermo Scientific, Waltham, MA), toluene (Thermo Scientific, Waltham, MA), methanol (MeOH, Thermo Scientific, Waltham, MA), ibuprofen (Thermo Scientific, Waltham, MA), SNAP-50 cartridges (Biotage, Uppsala, Sweden), thin layer chromatography paper (Thermo Scientific, Waltham, MA), prednisolone 21-hemisuccinate sodium salt (Sigma, St. Louis, MO), and hydrocortisone 21-hemisuccinate sodium salt (Sigma, St. Louis, MO).

The following instruments have been used for this study: Bruker 400 with BBO broadband probe and 60 sample auto express autosampler (Bruker, Billerica, MA), MESO QuickPlex SQ 120 MESO QuickPlex SQ 120 (Meso Scale Discovery, Rockville, MD), Agilent Cytation 5 cell imaging multimode reader (Agilent Technologies Inc., Santa Clara, CA), Agilent 1,260 Infinity II HPLC system (Agilent Technologies Inc., Santa Clara, CA) with UV-Vis DAD, paired with Wyatt miniDAWN MALS and Optilab dRI detectors (Wyatt Technologies, Santa Barbara, CA), Malvern Zetasizer Ultra zeta-potential analyzer with folded capillary zeta cells (Malvern Panalytical, Westborough, MA), Agilent Cary 100 UV-Vis spectrophotometer (Agilent Technologies Inc., Santa Clara, CA), and Millicell ERS-2 voltohmmeter (MilliporeSigma, Burlington, MA).

### 2.2 Synthesis of carboxymethylated HA (CMHA)

HA was derivatized with carboxymethyl functionalities to increase the conjugation sites available for the covalent attachment of molecules containing primary amines as previously published (Arrigali and Serban, 2022). Briefly, HA (2 g) was added to NaOH (20 mL, 45% w/v) and allowed to activate at room temperature (RT) for 2 h. In parallel, iodoacetic acid (2 g) was dissolved in isopropanol (50 mL). The viscous HA solution was added to isopropanol (150 mL), then the iodoacetic acid solution was added to the activated HA/isopropanol solution. The reaction mixture was allowed to react for 2 h and was then filtered using a Buchner funnel with a #2 Whatman filter paper. The white filter cake obtained after filtration was dissolved in deionized water (200 mL) and the pH of the resulting solution was neutralized using HCl (6 N). The resulting CMHA solution was then loaded into 3500 MWCO dialysis cassettes and dialyzed for 72 h with a minimum of three water changes every 24 h to remove residual reagent and salts. After dialysis, the CMHA solution was removed from the cassettes, frozen in a −80 °C freezer for a minimum of 4 h, and subsequently lyophilized. The reaction yielded 1.580 g of CMHA. Carboxymethylation was confirmed by proton nuclear magnetic resonance (^1^H-NMR).

### 2.3 Synthesis of amine modified ibuprofen

**FIGURE 1:**
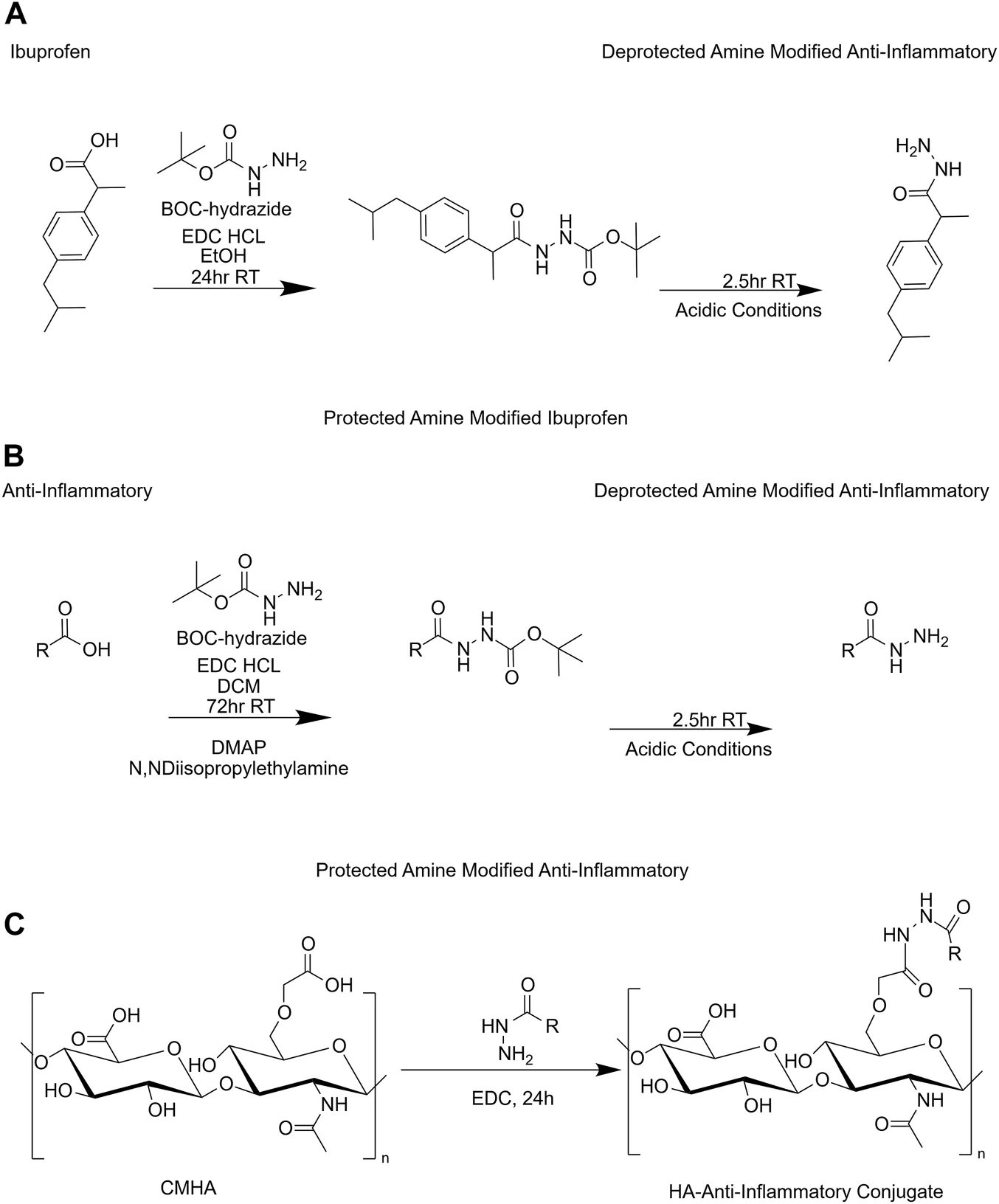
Reaction scheme for synthesis of HACs. **(A)** Amine functionalization used for ibuprofen. **(B)** Amine functionalization of carboxyl containing anti-inflammatories. **(C)** HA-anti-inflammatory conjugation reaction. BOC, tert-butyloxycarbonyl protecting group; CMHA, carboxymethyl-hyaluronic acid; DCM, dichloromethane; DMAP, 4-dimethylaminopyridine; EDC HCL, 1-(3-dimethylaminopropyl)-3-ethylcarbodiimide hydrochloride; EtOH, ethanol; HA, hyaluronic acid; R, rest of molecule; RT, room temperature.

IBU (Supplementary Figure S1) was functionalized with a primary amine in order to undergo a future carbodiimide reaction with CMHA (Figure 1A). IBU (3.4 g), EDC (3.84 g), and t-butyl carbazate (2.6 g), were added to a 200 mL round bottom flask. EtOH (50 mL) was added and the reaction was covered and allowed to stir at RT for 24 h. Then the reaction was concentrated *in vacuo* at 40°C to remove the EtOH. A ^1^H-NMR in D_6_ DMSO was performed on the protected amine modified IBU. The following day a deprotection of the amine was performed with the 5:1 ratio of DCM to TFA quenching after 2.5 h with a 1:1 ratio of toluene to MeOH and concentrated *in vacuo*. After drying, a ^1^H-NMR in D_6_ DMSO was performed.

### 2.4 Synthesis of amine modified hydrocortisone

Hydrocortisone 21-hemisuccinate sodium salt (Supplementary Figure S1) was functionalized with a primary amine in order to undergo subsequent conjugation with CMHA (Figure 1B). Hydrocortisone 21-hemisuccinate sodium salt (1.5 g), EDC (768 mg), DMAP (480 mg), a catalyst, and t-butyl carbazate (530 mg), a hydrazine derivative, were added to a 200 mL round bottom flask. DCM (25 mL) was then added followed by N,N-diisopropylethylamine (1.1 mL). The reaction was then covered and allowed to stir at RT for 72 h. Thin layer chromatography was performed to ensure the reaction was complete. Ethyl acetate was used for extraction after neutralizing with ammonium chloride. The organic and aqueous phases were then separated with a separatory funnel and sodium sulfate was added to the organic phase prior to vacuum filtration. The filtrate was placed in a clean round bottom flask and concentrated *in vacuo* at 40°C to remove ethyl acetate. The following day deprotection of the amine was performed with the 5:1 ratio of DCM to TFA quenching after 2.5 h with a 1:1 ratio of toluene to MeOH and concentrated *in vacuo*. After drying, purification was performed using flash chromatography. A Biotage SNAP 50 g cartridge with an initial gradient of 1% MeOH and 99% DCM and ending gradient of 10% MeOH was used for purification. To determine the final product a combination of thin layer chromatography and ^1^H-NMR were performed.

### 2.5 Synthesis of amine modified prednisolone

The same synthesis reaction was followed as outlined in Section 2.4 using the stoichiometric equivalent amount of prednisolone 21-hemiscuccinate sodium salt (Supplementary Figure S1) in place of hydrocortisone 21-hemisuccinate sodium salt.

### 2.6 Synthesis of HA conjugates

Conjugation of the anti-inflammatory molecules to CMHA were carried out in a similar manner as the previously published protocols (Arrigali and Serban, 2022). Briefly, CMHA (275 mg) was dissolved in nanopure water (30 mL). After CMHA was fully solubilized, EDC (183 mg) was added. While EDC solubilized, the amine modified small molecules (275 mg) were solubilized in EtOH (0.5 mL each) with sonication. The solubilized small molecule was added dropwise with a hypodermic needle very slowly. The water volume was increased stepwise to 90 mL followed by 150 mL, and the stir plate was increased to 400 rpm to prevent precipitation. The reaction was allowed to stir for 24 h at RT. After 24 h, the solution was filtered using a Buchner filter with # 2 Whatman paper. The filtrate was then neutralized to a pH of 7.0 and placed in 70 mL dialysis cassettes with a MWCO of 3,500 kDa. The solutions were dialyzed for 72 h with a minimum of three water changes per day before being placed in the freezer for 4 h and lyophilized. IBU-CMHA conjugate (I-HA) yielded 277.38 mg, PS-CMHA conjugate (P-HA) yielded 239.24 mg and HC-CMHA conjugate (HC-HA) yielded 278.5 mg of lyophilized product. The structure of the product was then assessed via ^1^H-NMR solubilized in D_2_O.

### 2.7 Determination of HAC physicochemical properties

Refractive index increment (dn/dc) and size exclusion chromatography multi-angle light scattering (SEC-MALS) were used to determine I-HA molecular weight and polydispersity index using methods previously described in detail (Veit et al., 2023). Potassium phosphate buffer (0.01 M, pH 7.58) was used as the mobile phase. For dn/dc, five concentrations (0.1–1.0 mg/mL) were injected into a Wyatt Opitlab RI detector and dn/dc was determined using manufacturer’s software analysis. SEC-MALS used Wyatt miniDAWN and Optilab detectors connected to an Agilent 1,260 Infinity II HPLC. I-HA was injected (50 μL, 0.4 mg/mL) at a flow of 1.0 mL/min through a 6.0–10,000 kDa 8 μm PL Aquagel-OH Mixed-H 7.5 × 300 mm SEC column (Agilent, PL1149-6800). Molecular weight and polydispersity were computed and reported by Wyatt ASTRA software.

Zeta-potential of the conjugates (5 mg/mL in nanopure water) was determined using a Malvern Zetasizer Red Ultra zeta-potential analyzer according to manufacturer’s instructions.

Estimated conjugation efficiency of HACs was determined using the relative absorbance contribution of individual conjugated components to the final conjugate. Specifically, the absorbance spectra of the HACs and each individual component of the conjugates (CMHA, anti-inflammatories, amine modified anti-inflammatories) at various concentrations was determined. Unique absorbance maxima with minimal crossover between components (I-HA, 222 nm; P-HA, 278 nm; HC-HA 280 nm) were used to determine the extinction coefficients for each component and the HACs. Where A = Absorbance, C = concentration, *ε* = extinction coefficient, H = HAC, i = conjugate component 1, and ii = conjugate component 2; and the following assumptions are true: A=C×ε (assuming path length is consistent), $C_{H}=C_{i}+C_{ii}$ , and $A_{H}=A_{i}+A_{ii}$ ; the following equation can be derived and was used to estimate the concentration of a component in a known concentration of conjugate: $C_{ii}=\frac{C_{H}\left(\varepsilon_{H}-\varepsilon_{i}\right)}{\left(\varepsilon_{ii}-\varepsilon_{i}\right)}$ . Using this method, the estimated conjugation efficiency of I-HA is 12.0%–12.9% w/w (mass of anti-inflammatory to total conjugate mass), P-HA is 9.0%–13.9%, and HC-HA is 7.6%–18.0%. While this method does rely on certain fallible assumptions, we currently lack the capability of a more certain process in determining conjugation efficiency for these conjugates and believe the method to provide a general approximation of conjugation efficiency. Further study into these HACs would require the development of a more robust method for determining conjugation efficiency. Absorbance was measured in 1 cm cuvettes on a Cary 100 UV/Vis spectrophotometer all samples were solubilized in a solution of 10% methanol and 90% phosphate buffer (0.01 M, pH 7.58) which permitted all components to remain soluble in tested linear absorbance range.

### 2.8 Cell culture

RAW 264.7 mouse macrophage cells were cultured in DMEM high glucose w/L-glutamine w/o sodium pyruvate with 10% FBS in a 37°C, 5% CO_2_, humidified incubator. Cells were grown in T75 flasks and media was replaced every 2–3 days until they reached 80% confluence. Cells were scraped gently from the flask using a sterile cell scraper and counted with an Invitrogen Countess II automated cell counter before plating.

House Ear Institute-organ of Corti 1 (HEI-OC1) cells were cultured in T75 flasks using DMEM supplemented with 10% FBS. The cells were grown until they reached approximately 80% confluence. HEI-OC1 cells were grown in a 33°C, 10% CO_2_, humidified incubator to support optimal cell growth. Cells were released with trypsin/EDTA and counted for plating.

After passing, HEI-OC1 cells (6×10^3^ cells, 100 µL/well) and RAW264.7 macrophages (1.5 × 104 cells, 100 µL per well) were seeded in 96-well plates and incubated overnight before treatments were started according to specific experimental conditions. The seeding densities and incubation conditions of both cell types were kept the same across all the experiments in this study unless specified otherwise.

### 2.9 Cell viability assays

After the indicated treatment for each experiment, MTS and LDH cytotoxicity assays were performed per the manufacturer’s protocols. Cells were treated with the indicated treatment in growth media (100 µL/well). For the LDH assay, 45 min prior to the end of treatment, lysis buffer (10 µL) was added to the lysis control wells, and all other groups were given sterile water (10 µL). The plate was tapped to mix then returned to the incubator for 45 min. Cell supernatant (50 µL) from all wells was then collected and, in a separate 96-well plate, added to LDH detection buffer (50 µL). After incubating at room temperature (30 min), stop solution (50 µL) was added, mixed, and the absorbance (490 nm, ref. 680 nm) was read on a microplate reader. All values were reference absorbance and blank (no cells) corrected, then normalized to the lysis control group (=100% lysis). For the MTS assay, treatment was removed, then MTS reagent (20 µL) in media (100 µL) was added to each well and the cells were returned to the incubator for 1.5 h. Absorbance was read then at 450 nm. All values were blank (no cells) corrected then normalized to the untreated controls.

### 2.10 Lipopolysaccharide stimulation of macrophages

Macrophages were plated as outlined above in Section 2.8. To determine optimal lipopolysaccharide (LPS) concentration for robust release of pro-inflammatory cytokines, macrophages were treated with a range of LPS concentrations (between 0–50 ng/mL) for 4 or 24 h. After treatment, culture supernatant was gently collected and analyzed for TNF-α and IL-6 release in the previously detailed ELISA kits according to the manufacturer’s instructions. The remaining cell supernatant was gently removed and cell viability assays were performed.

After selecting 10 ng/mL LPS as the standard LPS-stressed treatment for all subsequent experiments herein, the effect of this dose on the expression on ten inflammatory cytokines was evaluated using an MSD V-Plex Proinflammatory Panel per manufacturer’s protocols.

### 2.11 Screening of anti-inflammatory conjugates

Macrophages were prepared as outlined above in Section 2.8. The growth media was removed, and the cells were treated with HACs (1.5 mg/mL, 100 µL/well) or the estimated equivalent concentrations of unconjugated anti-inflammatory drugs for 4 h and 24 h. All groups, including controls, received 10 ng/mL LPS. After treatment, cell supernatant was collected and stored at −20°C until use. The most prominently released inflammatory cytokines (TNF-α and IL-6) were analyzed using the previously detailed ELISA kits according to manufacturer protocols.

### 2.12 Effect of I-HA treatment on macrophage cytokine release

Macrophages were cultured as described (Section 2.8). Growth media was aspirated, followed by the addition of a 100 μL of each treatment: LPS at 10 ng/mL; I-HA at 1.5 mg/mL with LPS at 10 ng/mL; or the estimated equivalent concentration of unconjugated IBU (0.18 mg/mL) with LPS at 10 ng/mL. After 24 h, 50 μL of cell supernatant was collected stored at −20°C until use. The samples were assayed using the MSD V-Plex proinflammatory panel according to manufacturer’s instructions.

### 2.13 Conditioned media preparation and HEI-OC1 treatment

T-75 tissue culture treated flasks were seeded with 10^6^ macrophages and allowed to incubate for 48 h before starting treatments, receiving fresh media after 24 h. All treatment flasks were given 15 mL of treatment in growth media. The conditioned media (CM) group received media alone. The LPS CM group was treated with 10 ng/mL LPS. Additionally, two control flasks were maintained without cells, one with media alone (control) and one with 10 ng/mL LPS (LPS control). After incubating for 24 h, the media from all the groups was collected, filtered with 0.22 µm syringe filters, and stored at −20°C.

HEI-OC1 cells were then prepared in 96-well plates (see Section 2.8). After discarding the growth media, the cells were treated with 100 µL of the treatments prepared above (control, LPS control, CM, and LPS CM). Following a 24 h treatment, LDH and MTS assays were performed (see Section 2.9).

### 2.14 Defined cytokine media treatment on HEI-OC1 cells

The four cytokines with the highest release from LPS-stressed macrophages were used to prepare defined cytokine blends. TNF-α, IL-6, IL-10, and IL-12p70 at 42, 16, 1.3, and 2.1 ng/mL, respectively, were denoted as the “High Inflammation” blend, which reflects their release concentrations in LPS-stressed macrophages (from Section 2.13). The “Reduced Inflammation” blend (17.8, 3.4, 0.2, and 2.1 ng/mL of each cytokine, respectively) reflects the decreased cytokine levels seen in LPS-stressed macrophages treated with I-HA (Section 2.12).

HEI-OC1 cells were cultured as outlined (Section 2.8 **)** and treated with 100 µL/well of the defined cytokine blends described above. After 24 h, MTS and LDH assays were performed as described (Section 2.9).

### 2.15 TM and RWM model compatibility and permeation testing

*In vitro* TM and RWM permeation models were previously developed by our group (Veit et al., 2022a; Singh et al., 2022), and grown as described in detail in these studies. TM models were grown with primary neonatal human keratinocytes cultured at an air-liquid interface for 11 days before testing. RWM models were grown with primary human small airway epithelial cells cultured at an air-liquid interface for 14 days. Permeation testing was also performed as described in the studies. Briefly, tissues were mounted in custom 3D printed permeation devices and transepithelial electrical resistance (TEER) was measured with a Millicell ERS-2 voltohmmeter to ensure tissue integrity. Tissues were placed in a 12-well plate containing 0.75 mL DPBS (receiver solution) and 0.1 mL of treatment in DPBS was placed onto the apical surface of the tissue. I-HA were treated at 20 mg/mL and IBU was treated at the estimated equivalent concentration of unconjugated IBU (2.4 mg/mL, applied as suspension due to solubility limit of ∼0.3 mg/mL). After being allowed to incubate in a 37°C humidified incubator for the indicated treatment time, receiver solution was collected and stored at −20°C until analysis.

I-HA concentration was determined using SEC-MALS/RI as detailed above (Section 2.7) against a standard curve. IBU concentration was determined using the same run conditions as SEC-MALS, but rather than use RI, the AUC of UV absorbance at 223 nm was used and compared to an IBU standard curve. The lack of this UV signal in the I-HA samples was also used to confirm that IBU had not noticeably detached from I-HA following tissue permeation.

Tissue viability was performed by placing 0.1 mL of the indicated treatments in DPBS onto the surface of each tissue. A 5% w/v SDS solution was used as a cytotoxic control. The tissues were then placed in fresh growth media in a 37°C, 5% CO_2_, humidified incubator for 24 h. After treatment, the tissue was thoroughly washed with DPBS then placed into 0.3 mL media containing MTT (1 mg/mL), which is reduced to a formazan by living cells, for 3 h in the incubator. The tissues are then placed into a new plate, submerged in 2 mL isopropanol to dissolve the formazan, sealed, and placed on a shaker for 2 h. Absorbance at 570 nm is then measured in technical duplicates on a Cytation 5 plate reader. TEER and histological preparation/analysis of treated tissues was performed as previously described in detail (Veit et al., 2022a; Singh et al., 2022).

### 2.16 Statistical analysis

Prism version 10 (GraphPad, San Diego, CA) was used for normality testing and statistical analysis. Each figure caption details the specific tests utilized for each dataset. A *p*-value of <0.05 was considered statistically significant.

## 3 Results

### 3.1 HAC synthesis

**FIGURE 2:**
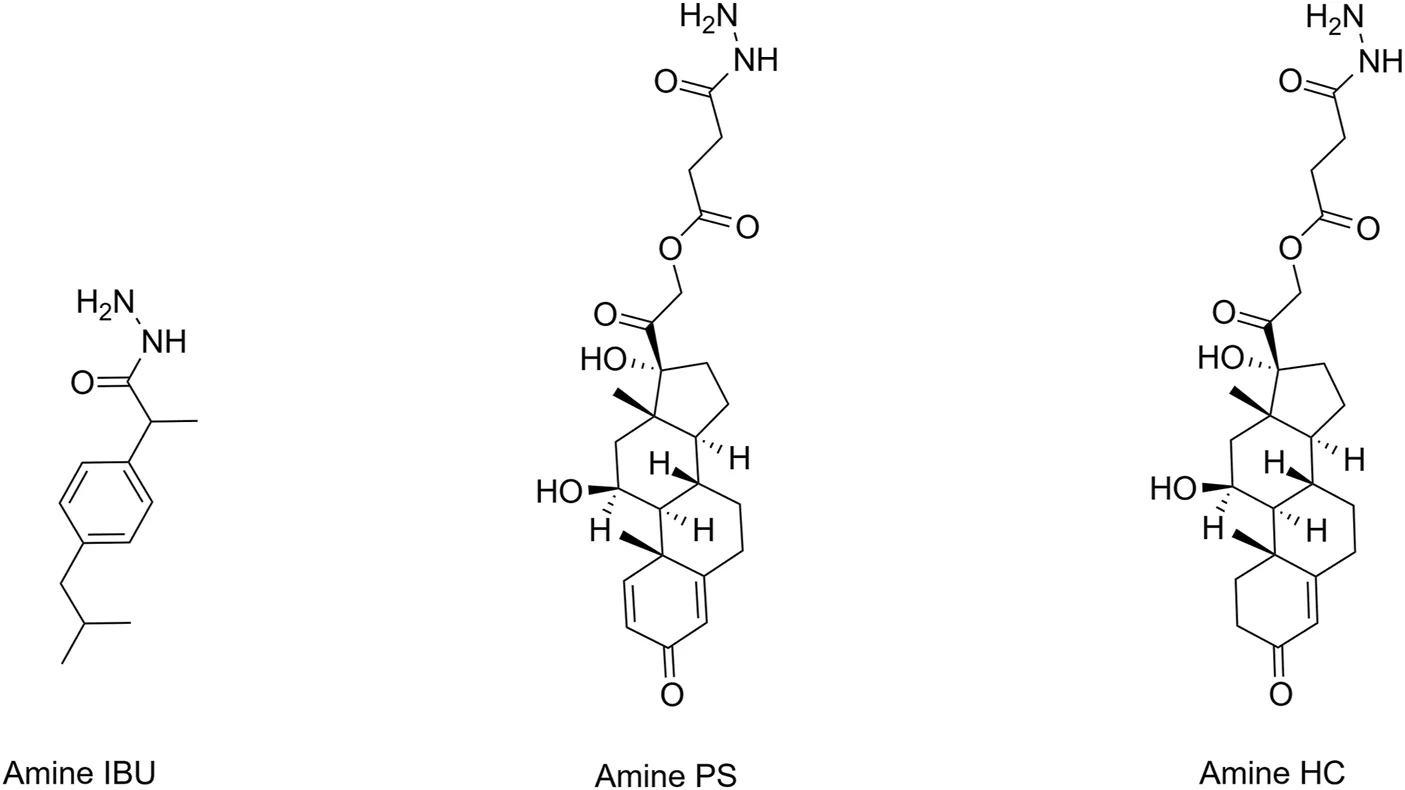
Structures of aminated anti-inflammatory molecules. HC, hydrocortisone; IBU, ibuprofen; PS, prednisolone.

Three anti-inflammatory drugs; IBU, PS, and HC, were selected for conjugation to HA (Supplementary Figure S1). The initial step of the synthesis process was to enrich HA with carboxyl moieties to increase drug conjugation sites. The structure of this intermediate (CMHA) was confirmed with ^1^H-NMR, which also revealed a carboxymethylation efficiency of 52%–57% (% of possible binding sites). Separately, the anti-inflammatory drugs were each functionalized with a primary amine to facilitate subsequent conjugation to HA (Figure 2). Successful modification was confirmed via ^1^H-NMR (Supplementary Figures S2A–C), by the additional peaks seen at approximately 1.5 ppm, which originate from the addition of the BOC moiety. The aminated anti-inflammatory drugs were then covalently conjugated to HA, and the successful syntheses of the three resulting HACs, denoted as I-HA, P-HA, and HC-HA (IBU, PS, and HC, respectively), were confirmed via ^1^H-NMR (Supplementary Figures S3A–C) by the additional peak seen at ∼ 2.8 ppm. All three conjugates were also found to be significantly more water soluble (greater than 20 mg/mL in DPBS) than their unconjugated equivalents.

### 3.2 Anti-inflammatory screening of HACs

To assess the anti-inflammatory properties of the conjugates, cytokine release was evaluated in LPS-stimulated macrophages with and without treatments. First, the optimal LPS dose that elicited a robust release of TNF-α and IL-6 (Supplementary Figures S4A,B1), without affecting cell viability (Supplementary Figure S4C), was determined. Based on these results, LPS was dosed at 10 ng/mL in all subsequent assays. A panel of additional inflammatory cytokines was then evaluated to profile the cytokine release patterns of macrophages in response to LPS-stress (Supplementary Figure S5). This found that TNF-α and IL-6 were the predominant cytokines released, and showed that LPS induced a very strong response relative to control in every other cytokine tested, with the exception of IFN-γ, which was below the quantification limit in both groups.

**FIGURE 3:**
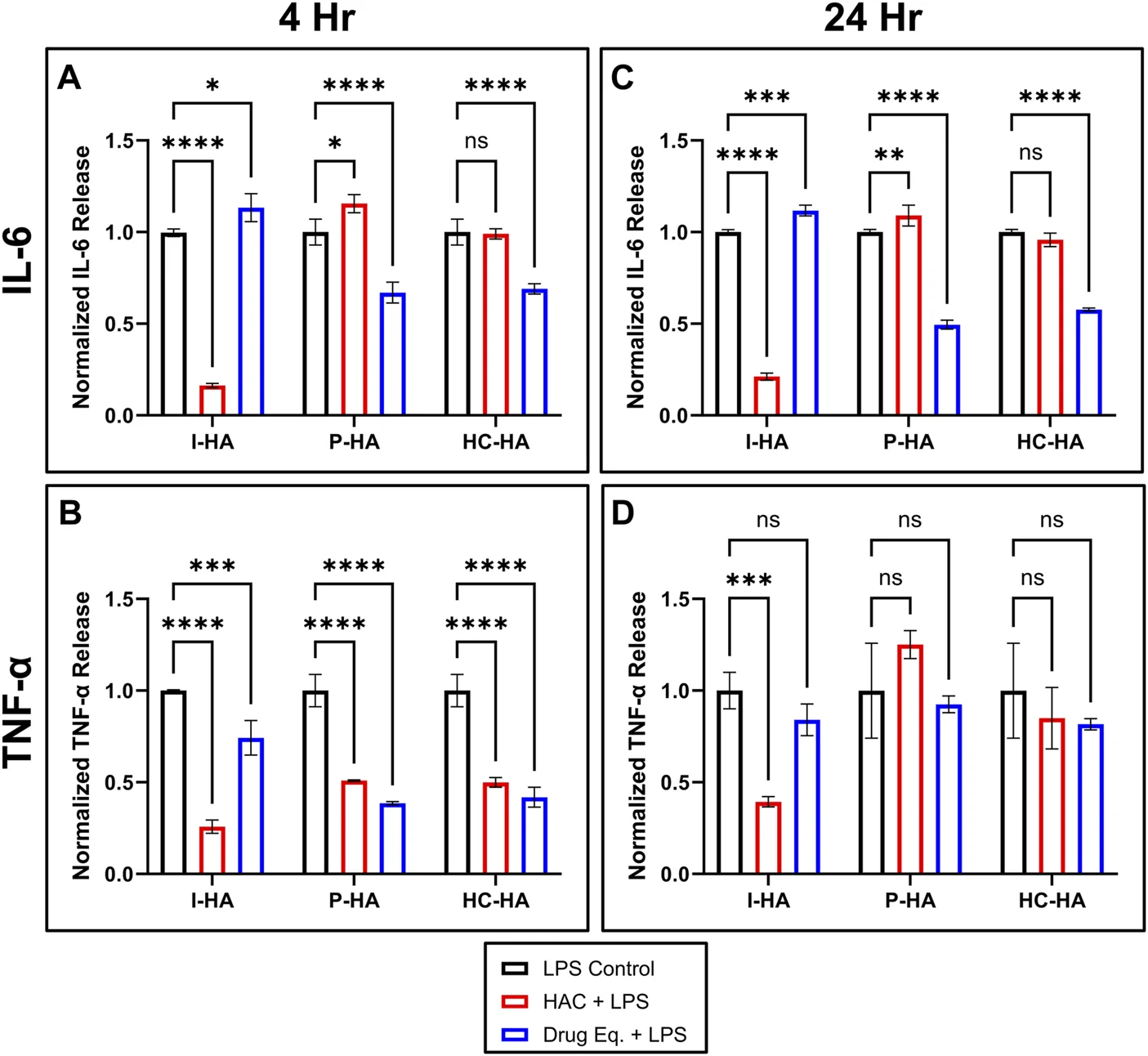
Normalized cytokine release in LPS-stressed macrophages treated with HACs, or the respective unconjugated anti-inflammatory drugs. **(A)** IL-6 and **(B)** TNF-α release from RAW264.7 macrophages treated for 4 h with 10 ng/mL LPS (all) and simultaneously, untreated control (LPS Control, black), 1.5 mg/mL of the indicated HAC (HAC + LPS, red), or the estimated equivalent concentration of unconjugated anti-inflammatory drug (Drug Eq. + LPS, blue). **(C)** IL-6 and **(D)** TNF-α release from RAW264.7 macrophages treated for 24 h with 10 ng/mL LPS (all) and simultaneously, untreated control (black), 1.5 mg/mL of the indicated HAC (red), or the estimated equivalent concentration of unconjugated anti-inflammatory drug (blue). **(A–D)** Values shown are normalized to the level of the respective untreated controls. n = 3 (I-HA); n = 2–4 (P-HA, HC-HA); two-way ANOVA with Dunnett’s Correction; *p < 0.05, **p < 0.01, ***p < 0.001, ****p < 0.0001, ns = not significant. All graphs show mean ± SD. HAC, hyaluronic acid-anti-inflammatory conjugate; HC-HA, hydrocortisone-HA conjugate; I-HA, ibuprofen-HA conjugate; LPS, lipopolysaccharide; P-HA, prednisolone-HA conjugate.

Based on these findings, the anti-inflammatory potential of the three HACs was evaluated in the two most responsive cytokines (TNF-α and IL-6). While unconjugated PS and HC reduced IL-6 release in both the 4 and 24 h treatment condition, reduction of TNF-α was only observed in the 4 h condition (Figure 3). Similarly, P-HA and HC-HA also showed a reduction in TNF-α in the 4 h condition; however, this was to a lesser extent than the unconjugated drugs. Unlike the unconjugated drugs, neither P-HA nor HC-HA reduced IL-6 release in either condition. Conversely, I-HA significantly decreased IL-6 and TNF-α release in both the 4 and 24 h treatment conditions. In this case, unconjugated drug (IBU) only decreased TNF-α release in the 4 h condition, and did so to a lesser extent than the conjugate (I-HA). Based on these results, P-HA and HC-HA were not further investigated, as they significantly underperformed relative to unconjugated drug. Therefore, I-HA was selected as the lead candidate for further investigation.

### 3.3 I-HA characterization

#### 3.3.1 Physicochemical properties

Various physicochemical properties of I-HA were then evaluated to provide additional information into properties which can be used to optimize future formulations work, and to provide an estimate of drug conjugation efficiency. Our analyses showed that I-HA has an average molecular weight of 27.8 kDa, a polydispersity index of 1.399, a refractive index increment of 0.1495 mL/g, and a ζ-potential of −26.77 mV (Supplementary Table S1). The estimated conjugation efficiency of I-HA was determined to be 12.0%–12.9% w/w (mass I to total mass I-HA).

#### 3.3.2 Cytocompatibility

**FIGURE 4:**
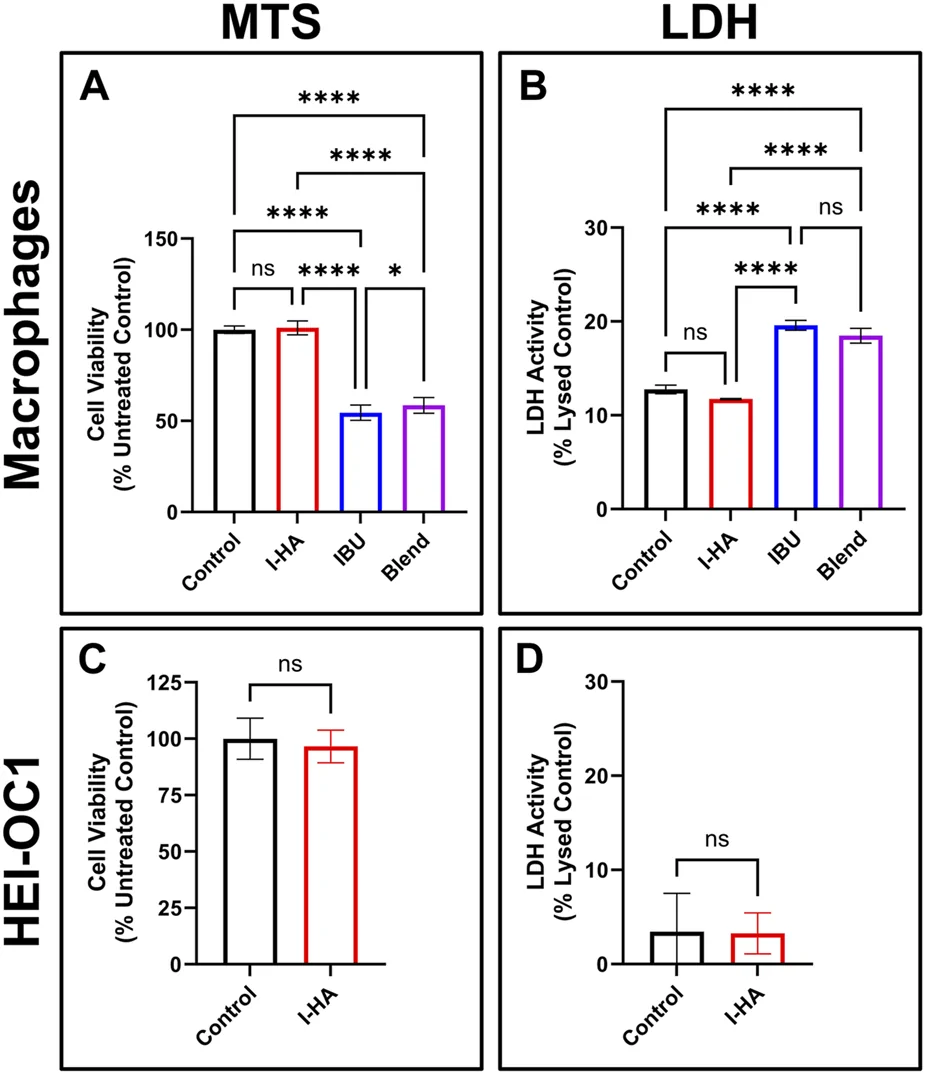
I-HA cytocompatibility in macrophages and cochlear cells. **(A)** MTS and **(B)** LDH cell viability assay in RAW264.7 macrophages treated for 24 h with control (black), 1.5 mg/mL I-HA (red), the estimated equivalent concentration (0.18 mg/mL) of unconjugated IBU (blue), or a blend (purple) of the estimated equivalent concentration of unconjugated IBU (0.18 mg/mL) and CMHA (1.32 mg/mL). **(A)** n = 12; **(B)** n = 4; **(A, B)** one-way ANOVA with Tukey’s Correction; *p < 0.05, ****p < 0.0001, ns = not significant. **(C)** MTS and **(D)** LDH cell viability assay in HEI-OC1 cells treated for 24 h with control (black) or 1.5 mg/mL I-HA (red). **(C, D)** n = 9; *t*-test; ns = not significant. All graphs show mean ± SD. I-HA, ibuprofen-HA conjugate; LDH, lactate dehydrogenase.

To confirm I-HA has a minimal risk of cytotoxicity, cytocompatibility assays were performed in macrophages and HEI-OC1 cochlear cells. In macrophages, 24 h I-HA treatment showed no signs of decreased cell viability (Figure 4A) or membrane integrity (Figure 4B) relative to untreated control. Conversely, unconjugated IBU, or a blend of CMHA and unconjugated IBU (at the estimated equivalent concentrations), severely reduced cell viability (55% and 59%, respectively) and membrane integrity (20% and 19% cell lysis, respectively). A dose-response assay showed that IBU had an IC_50_ (concentration leading to 50% cell viability) of 0.135 mg/mL (Supplementary Figure S6A), despite the higher equivalent concentration of I-HA showing no loss in viability.

Similarly, in HEI-OC1 cells, no cell viability (Figure 4C) or membrane integrity (Figure 4D) issues were observed with I-HA treatment. A dose-response assay (Supplementary Figure S6B) showed that HEI-OC1 cells are less affected by IBU than macrophages, with an IC_50_ above IBU’s solubility limit (0.3 mg/mL), which resulted in only a 28% reduction in cell viability.

#### 3.3.3 I-HA inflammatory cytokine panel

**FIGURE 5:**
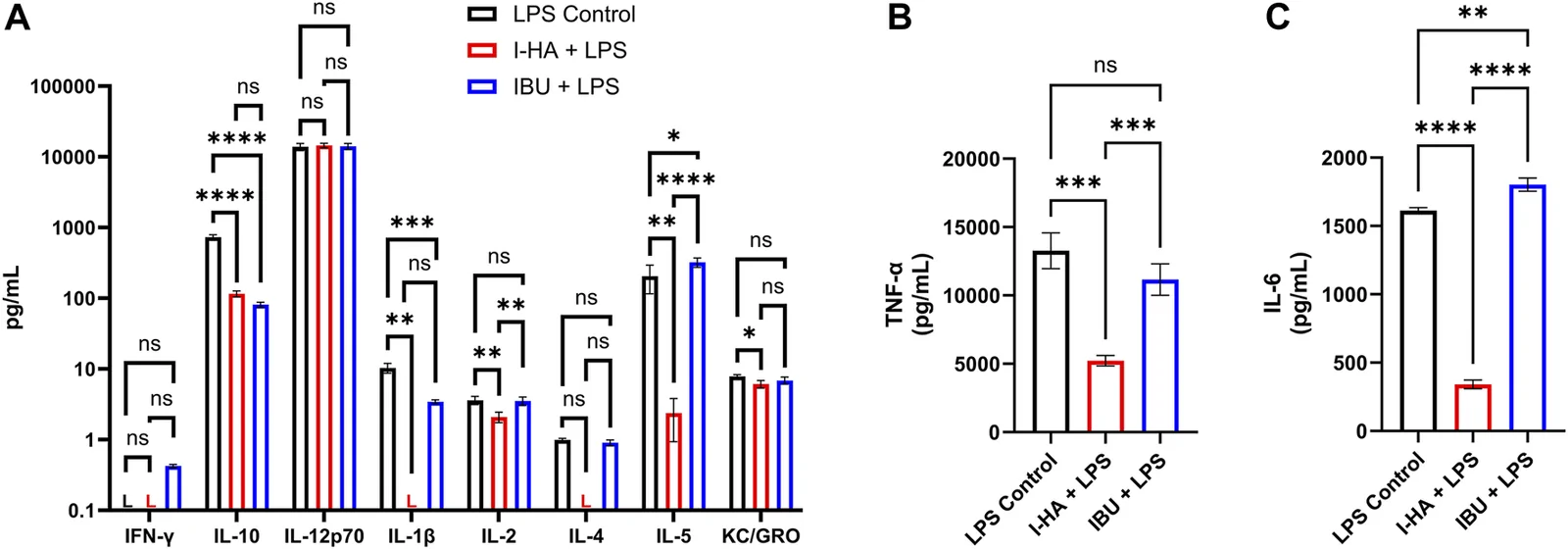
Cytokine release in LPS-stressed macrophages treated with I-HA or IBU. **(A)** Cytokine release in RAW264.7 macrophages treated for 24 h with 10 ng/mL LPS (all) and simultaneously, untreated control (LPS Control, black), 1.5 mg/mL I-HA (I-HA + LPS, red), or the estimated equivalent concentration (0.18 mg/mL) of unconjugated IBU (IBU + LPS, blue). **(B)** IL-6 and **(C)** TNF-α release, which were retested independently due to exceeding the upper quantification limit in the multiplex assay. **(A–C)** Mean ± SD; n = 3–4; an independent one-way ANOVA with Tukey’s Correction was performed for each cytokine, for cytokines with a group below the assay quantification limit (“L”) the valid groups were tested against the value of the quantification limit; *p < 0.05, **p < 0.01, ***p < 0.001, ****p < 0.0001, ns = not significant. I-HA, ibuprofen-HA conjugate; L, below lower detection limit; LPS, lipopolysaccharide.

An inflammatory cytokine panel was then used to evaluate the direct effects of I-HA on macrophage cytokine release. Relative to the LPS-stressed control, I-HA treatment reduced the levels of IL-10, IL-1β, IL-2, IL-5, KC/GRO (CXCL1), IL-6, and TNF-α (Figures 5A–C). IL-12p70 was not reduced by I-HA and IFN-γ was below the assay quantification limit in both I-HA and the control. I-HA did reduce IL-4 levels below the assay quantification limit; however, the control and IBU groups were not significantly different from the quantification limit. Conversely, IBU only reduced the release of IL-10 and IL-1β, and IL-10 was the only cytokine IBU reduced more than I-HA treatment. Additionally, IBU also significantly increased the levels of IL-5 and IL-6 relative to control.

#### 3.3.4 Inflammatory cytotoxicity in cochlear cells

**FIGURE 6:**
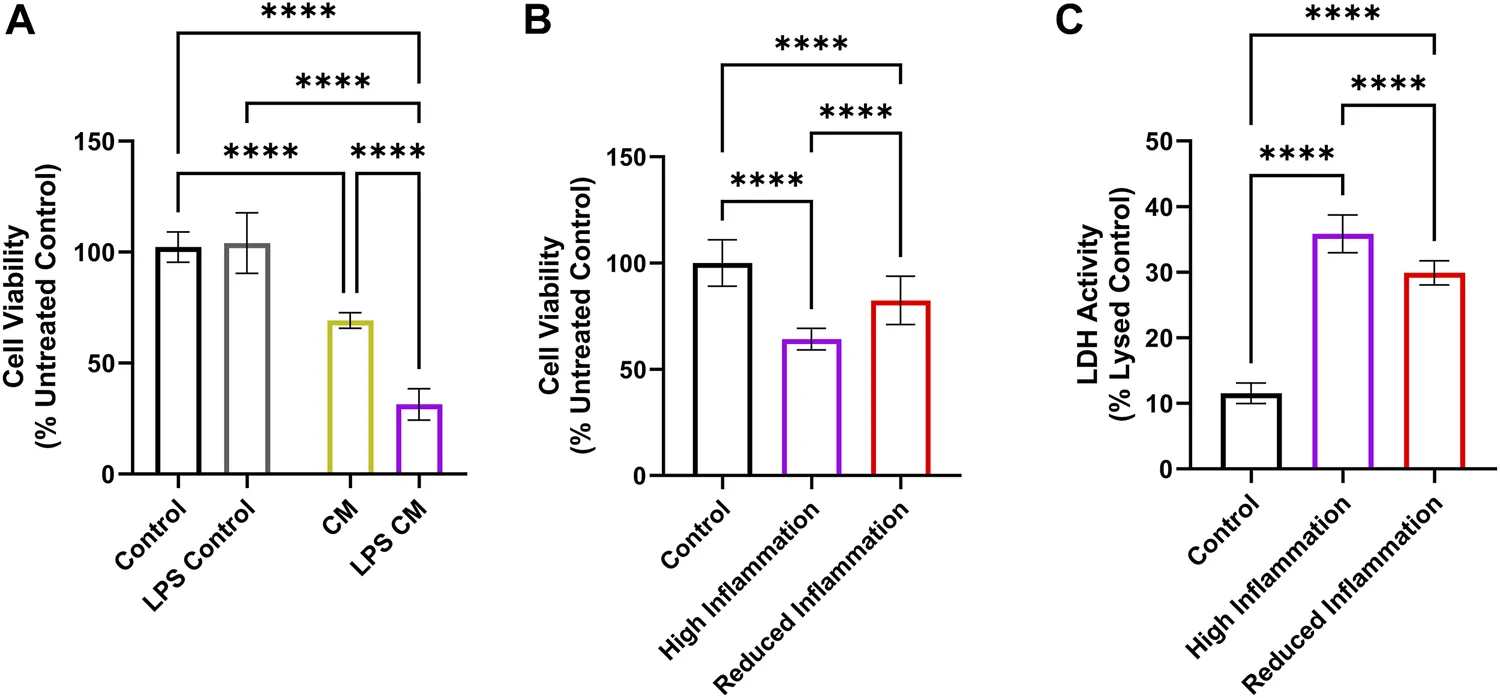
Effect of macrophage cytokine release on cochlear cell viability. **(A)** MTS viability assay in HEI-OC1 cells treated with media (Control, black), media with LPS (LPS Control, grey), or with conditioned media consisting of media collected from macrophages after 24 h (CM, gold) or media collected from macrophages treated with LPS for 24 h (LPS CM, purple). n = 8. **(B)** MTS and **(C)** LDH viability assays in HEI-OC1 cells treated with control (black), a blend of cytokines reflecting the cytokine levels released by LPS-stressed macrophages (“High Inflammation”, purple), or a blend of cytokines reflecting the cytokine levels released by LPS-stressed macrophages treated by 1.5 mg/mL I-HA (“Reduced Inflammation”, red). n = 16–40. **(A–C)** Brown-Forsythe and Welch one-way ANOVA test with Dunnett T3 Correction; ****p < 0.0001. All graphs show mean ± SD. CM, conditioned media; I-HA, ibuprofen-HA conjugate; LDH, lactate dehydrogenase; LPS, lipopolysaccharide.

To assess the effect of macrophage-mediated inflammation on cochlear cell survival, conditioned media (CM) and LPS CM were produced by treating macrophages for 24 h with media alone or media with LPS, respectively. Cochlear cell viability was significantly reduced by treatment with LPS CM compared to control (media only) and LPS control (media with LPS) (Figure 6A). However, CM also significantly reduced cell viability, although to a lesser extent than LPS CM. Since the CM (from unstressed macrophages) also resulted in cell death, it is unclear if the increased cytokine levels in LPS CM are solely responsible for the observed effect. To further understand this, defined cytokine blends were created with the four most prominent cytokines (TNF-α, IL-6, IL-10, and IL-12p70) released by LPS-stressed macrophages. One blend reflected the previously quantified (Supplementary Figure S5) cytokine concentrations found in LPS-stressed macrophages (denoted as “High Inflammation”; 42, 16, 1.3, and 2.1 ng/mL, respectively), and the other reflected the reduced cytokine concentrations found in LPS-stressed macrophages that were treated with I-HA (denoted “Reduced Inflammation”; 17.8, 3.4, 0.2, and 2.1 ng/mL, respectively). These blends were then applied to HEI-OC1 cells for 24 h, and the cell viability was determined. As expected, the “High Inflammation” blend significantly reduced cell viability (down to 64%) and membrane integrity (36% cell lysis) (Figures 6B,C). The “Reduced Inflammation” blend, reflective of I-HA treatment, showed a significant improvement in cell viability (82%) and membrane integrity (30% cell lysis) relative to the “High Inflammation” group. Moreover, investigating the effects of individual cytokines at the “High Inflammation” concentration did not show any negative effects on cell viability (Supplementary Figure S7), suggesting that the interaction between multiple cytokine pathways is critical to the cellular damage.

#### 3.3.5 TM and RWM models: I-HA permeation and tissue compatibility

**FIGURE 7:**
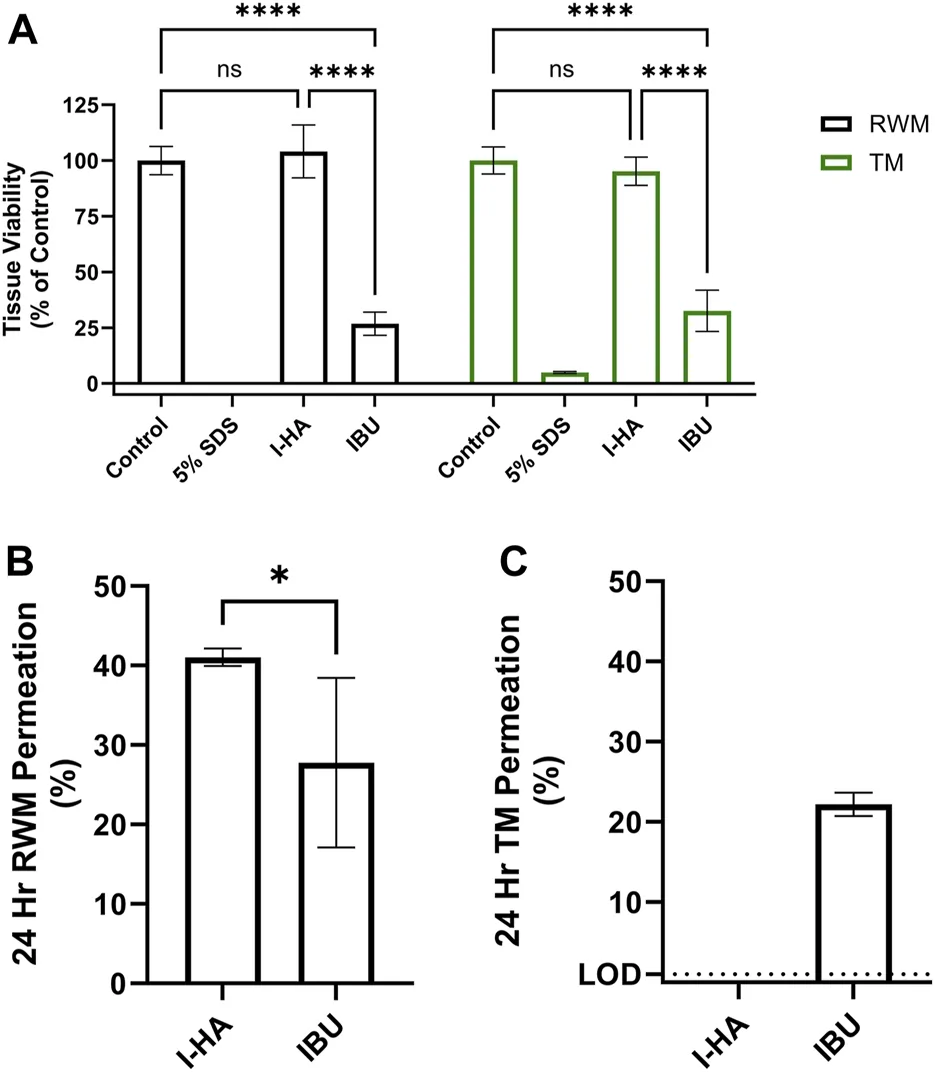
I-HA tissue compatibility and permeation across RWM and TM models. **(A)** RWM and TM tissue model viability after 24 h exposure to control (DPBS), cytotoxic control (5% SDS), I-HA (20 mg/mL), or the estimated equivalent concentration (2.4 mg/mL) of unconjugated IBU. Mean ± SD; n = 3–6; two-way ANOVA with Šídák Correction; ***p < 0.001, ****p < 0.0001, ns = not significant. **(B)** 24 h permeation of I-HA across *in vitro* RWM permeation model. n = 4–7; Welch’s *t*-test; *p < 0.05. **(C)** 24 h permeation of I-HA across *in vitro* TM permeation model. “LOD” dotted line indicates the approximate limit of detection of I-HA which was not detected in this experiment. n = 4. **(B, C)** Show percent of total drug permeated following application of 0.1 mL of I-HA (20 mg/mL) or estimated equivalent concentration (2.4 mg/mL) of unconjugated Ibu. Mean ± SD. I-HA, ibuprofen-HA conjugate; RWM, round window membrane; TM, tympanic membrane.

To evaluate I-HA’s potential as a topical therapeutic, tissue compatibility was assessed in vitro TM and RWM permeation models. As seen in the cytocompatibility assays, unconjugated IBU showed significant toxicity in both tissue models, while I-HA caused no apparent toxicity (Figure 7A).

**FIGURE 8:**
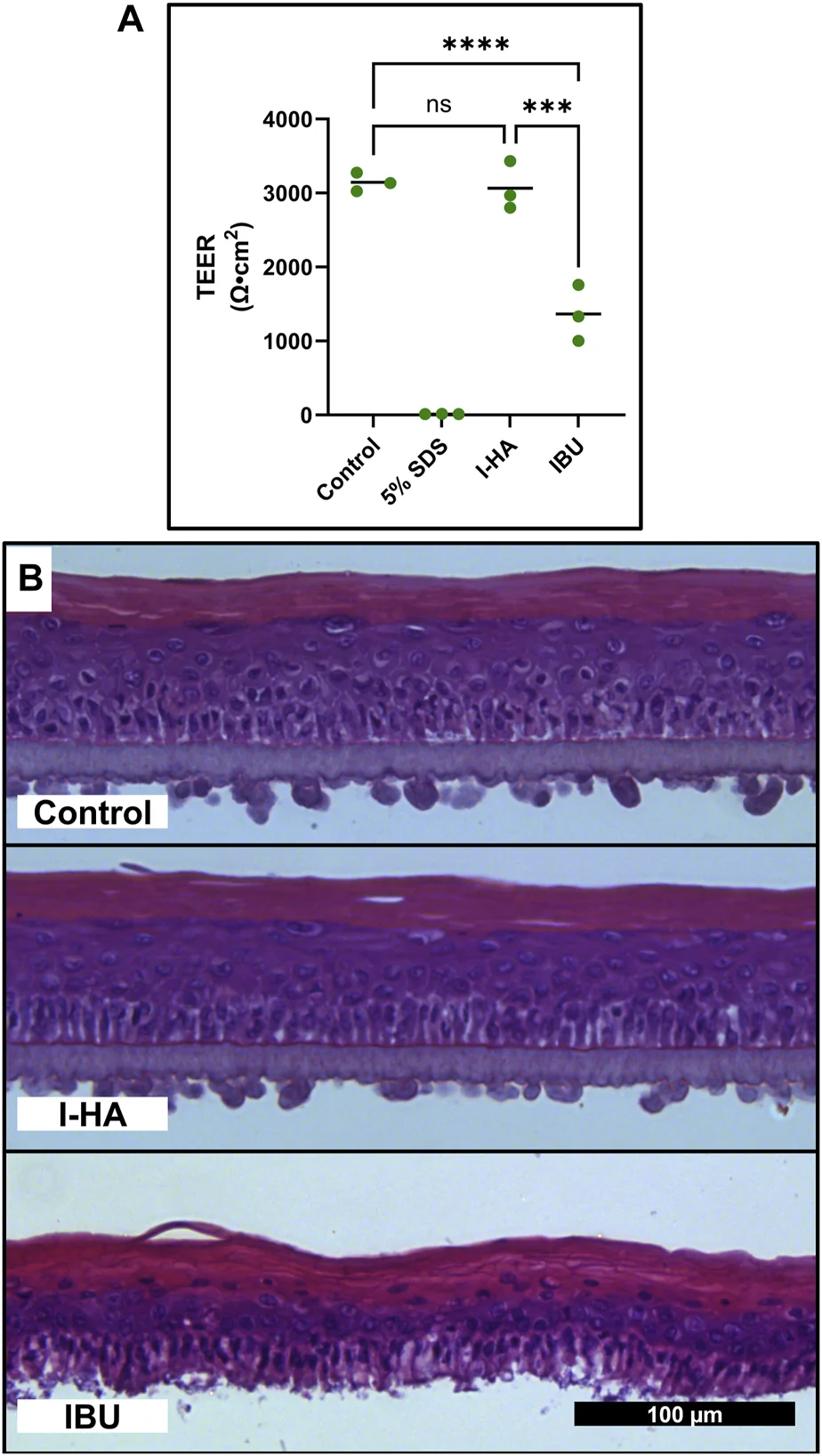
Cytotoxicity of IBU in TM tissue models. **(A)** TEER TM tissue models after 24 h exposure to control (DPBS), cytotoxic control (5% SDS), I-HA (20 mg/mL), or the estimated equivalent concentration (2.4 mg/mL) of unconjugated IBU. n = 3; two-way ANOVA with Šídák Correction; ***p < 0.001, ****p < 0.0001, ns = not significant. **(B)** Representative micrograph of H&E stained TM tissues exposed to control (DPBS), I-HA (20 mg/mL), or estimated equivalent concentration (2.4 mg/mL) of unconjugated IBU for 24 h. Scale bar applies to all panels. I-HA, ibuprofen-HA conjugate; SDS, sodium dodecyl sulfate; TEER, transepithelial electrical resistance; TM, tympanic membrane.

I-HA permeability across the RWM and TM models was then evaluated. After 24 h, 41% of the applied I-HA permeated the RWM model, while only 28% of an equivalent concentration of unconjugated IBU permeated (Figure 7B). The kinetics of this permeation was also evaluated to provide additional insight into I-HA flux over time (Supplementary Figure S8). When tested in the TM model, total I-HA permeated was below the method detection limit (Figure 7C). Lower TM permeability is expected, as it is known to be significantly less permeable than the RWM. Intriguingly, IBU was able to readily permeate the TM at levels comparative to the RWM model. To understand this, a loss of tissue integrity was then investigated as a possible explanation to the higher-than-expected permeability of IBU across the TM. For this, transepithelial electrical resistance (TEER), a measure of barrier integrity, was evaluated. The TEER values were significantly decreased by treatment with IBU, but not I-HA, confirming that IBU treatment resulted of loss of barrier integrity (Figure 8A). We further confirmed this histologically, with our analyses revealing that IBU treatment resulted in drastic changes to tissue stratification, keratinocyte differentiation (evidenced by the persistent nuclei within the granular and corneal layers), and basilar keratinocytes detachment (Figure 8B). In contrast, I-HA did not noticeably change tissue morphology.

## 4 Discussion

This study explores the development of potential topical ototherapeutics against inflammation-mediated cochlear cell damage. For this, we covalently attached several anti-inflammatory drugs to HA, with the intent to improve permeation across tissues (Shibata et al., 2012), cellular internalization (Arrigali and Serban, 2022), and therapeutic efficacy (Rogha and Kalkoo, 2017). PS, HC, and IBU were selected as anti-inflammatory drugs due to their widespread clinical use for the management of inflammatory conditions (Trune and Canlon, 2012; Taha et al., 2019; Paglia et al., 2021).

In terms of conjugation strategy, the intent was to have reaction partners that could be coupled via simple carbodiimide-mediated chemistry. To this end, HA was first enriched in carboxy functionalities (yielding CMHA), to increase the potential conjugation sites for the selected drugs. As an initial approach, we explored adding amine functionalities onto HA, with the intent of using the intrinsic carboxyl functionalities of the selected anti-inflammatory drugs for coupling. This strategy was unsuccessful as it led to HA crosslinking. Therefore, as an alternative approach, the anti-inflammatory drugs were chemically functionalized to contain primary amine groups. This was achieved by adding a BOC-protected hydrazine derivative, tert-butyl carbazate, which was subsequently deprotected to reveal the reactive amine. The reaction scheme for the BOC-coupling involved the use of a catalyst (DMAP), and led to successful generation of amine-PS and amine-HC, which were then effectively coupled to CMHA. However, for the I-HA conjugate synthesis, a reaction-specific DMAP byproduct posed purification challenges and inadequate coupling to CMHA. The final I-HA synthesis was instead performed without the use of DMAP, which still allowed for effective synthesis and downstream IBU conjugation. It is important to note that the HA conjugation also led to water soluble compounds, unlike the initial unconjugated drugs, which are only soluble in non-aqueous conditions. This is advantageous, as it is expected to translate to user- and manufacturing-friendly drug formulations.

The anti-inflammatory properties of the obtained conjugates were then evaluated. The screening assays developed for this purpose were designed to mirror physiological processes associated with acoustic trauma. Intense noise exposure has been reported to recruit and activate inflammatory cells, such as macrophages, in the cochlea. This results in the release of inflammatory cytokines including IL-6 and TNF-α, and contributes to cochlear damage (Wakabayashi et al., 2010; Nakamoto et al., 2012; Harrop-Jones et al., 2016; Landegger et al., 2019; Yoon et al., 2019). Therefore, the HACs were first screened in macrophages stressed by LPS, a commonly used inducer of inflammation (Liu et al., 2019; Yi et al., 2021; Chen et al., 2023). Our results indicate that I-HA was able to significantly reduce IL-6 and TNF-α release to a greater extent than an equivalent concentration of unconjugated IBU, and to a greater extent than all other tested HACs. Conversely, neither P-HA nor HC-HA were able to outperform the anti-inflammatory effects of their unconjugated forms, and in most cases, displayed no anti-inflammatory effects at all. Based on these results, P-HA and HC-HA were eliminated for further investigation.

The physicochemical properties of I-HA were then determined to inform on properties which could be important for subsequent formulation studies. The cytocompatibility of I-HA was also assessed in macrophages and cochlear cells. At anti-inflammatory concentrations, I-HA showed good cytocompatibility in both cell types. However, macrophages treated with the estimated equivalent concentration of unconjugated IBU showed significant cytotoxic effects. Since the unconjugated blend of IBU and CMHA was also cytotoxic, we eliminated blends from further cellular screening. In cochlear cells, cytotoxic effects from IBU treatment were still observed, but at higher concentrations than in macrophages. The observed cytotoxicity of IBU is not unexpected, as it is well-documented that IBU can be cytotoxic in many cells and organisms (Ngo and Bajaj, 2023). Other research also suggests that IBU may play a role in hearing loss following prolonged exposure (Curhan et al., 2012; Kyle et al., 2015), although evidence of permanent ototoxicity is lacking (Martín-Saldaña et al., 2018). Overall, our data may indicate that HA conjugation could greatly reduce or eliminate the detrimental effects of IBU, while still retaining the desired anti-inflammatory properties.

As a next step, I-HA was investigated in a larger panel of inflammatory cytokines. The results confirmed that in addition to TNF-α and IL-6, I-HA also reduces the release of several other relevant cytokines (IL-1β, IL-2, IL-5, and KC/GRO). The extent of this reduction was greater than observed for IBU, with the exception of IL-10, which I-HA reduces slightly less than IBU. Considering that TNF-α, IL-6, IL-1β, KC/GRO (i.e., CXCL1), and IL-2 are generally proinflammatory (So et al., 2007; Kim et al., 2014; Capobianco et al., 2016; Silva et al., 2017; Wu et al., 2022), our results suggest that I-HA would be significantly better at reducing inflammation than unconjugated IBU. On the other hand, IL-4 is typically anti-inflammatory (Chatterjee et al., 2014), and IL-5 can be pro- or anti-inflammatory depending on other factors. IL-10 is also considered anti-inflammatory (Chatterjee et al., 2014), and although I-HA did reduce IL-10 release, it did so to a lesser extent than IBU. Collectively, inflammation pathways and cytokine release patterns are complex processes which makes drawing certain conclusions challenging. However, the general patterns we observe in macrophages suggest that I-HA treatment can reduce inflammation markers, and do so to a significantly greater extent than IBU alone.

Together, the improvement in cytotoxicity and improved reduction in cytokine release of I-HA relative to unconjugated IBU, show that this chemical conjugation plays a critical role in the function of I-HA. This presents an interesting question into the mechanism of these differences, why this is the case, and what can be learned from this for future research. Although this study did not investigate these questions, we believe future studies should seek to elucidate the mechanisms responsible for these results.

To model the interplay between macrophage-mediated inflammation and damage to cochlear cells in response to acoustic stress, we designed an experiment where cochlear cells (HEI-OC1) were exposed to macrophage-conditioned media. A co-culture condition of the 2 cell types was considered; however, the cell lines require drastically different culture conditions which pose experimental and data interpretation challenges. Instead, we generated conditioned media by first incubating the macrophages with media, with and without LPS, then collected the resulting conditioned media (LPS CM and CM, respectively) to treat the HEI-OC1. When compared to control media, the LPS CM drastically reduced HEI-OC1 viability; however, CM also reduced HEI-OC1 viability, but to a lesser extent than the LPS CM. While this does support the assertion that macrophage stimulation is damaging to cochlear cells, the interpretation of the extent of the cell damage directly caused by inflammatory cytokine release solely based on these results was ambiguous. To further clarify this, we created defined cytokine blends consisting of the four most released cytokines (TNF-α, IL-6, IL-12p70, and IL-10) at concentrations found in stimulated macrophages (“High Inflammation”), and the cytokine levels in I-HA treated stimulated macrophages (“Reduced Inflammation”). Through this experiment, we were able to show that the “High Inflammation” blend significantly reduces cell viability, while the “Reduced Inflammation” blend, representative of I-HA treatment, significantly improved HEI-OC1 viability. These results further support I-HA’s potential to protect cochlear cells from inflammatory damage. Interestingly, when developing this cytokine blend, we found that, when used individually, neither of the four cytokines in the “High Inflammation” concentration decreased cell viability. This finding was not entirely unexpected due to the interdependent nature of complex inflammatory pathways. While HEI-OC1 cells are amongst the most commonly used cochlear cells lines in auditory research (Kalinec et al., 2016), the *in vitro* results presented here should be validated with future *in vivo* studies to account for the limitations of both *in vitro* and *in vivo* models in hearing research (Lee and Waldhaus, 2022).

Considering our intent to develop topical ototherapeutics, we then evaluated I-HA permeation across *in vitro* RWM and TM permeation models previously developed by our group (Veit et al., 2022a; Singh et al., 2022). Initial tissue toxicity screening shows that I-HA does not appear to be cytotoxic; however, as seen in cellular assays, IBU caused significant loss of tissue viability. Permeation testing indicates that I-HA was able to readily permeate the RWM model and do so more efficiently than unconjugated IBU. This increased capability in RWM permeation associated with the presence of HA has also been previously observed *in vitro* (Arrigali and Serban, 2022) and *in vivo* (Rogha and Kalkoo, 2017).

In testing permeability across the previously developed *in vitro* TM permeation model (Veit et al., 2022a), we were not able to detect I-HA levels above the limit of detection (indicating permeation is < ∼1% over 24 h). This result is expected, as the TM is significantly more challenging to permeate than the RWM due to the epidermal skin-like composition of its outer layer which contains a lipid rich stratum corneum, as well as tightly packed keratinocytes linked by tight junctions (Yang et al., 2017; Veit et al., 2022b). Surprisingly, we did see significant IBU permeation across the TM model, to a level that nearly matched the much more permeable RWM model. We hypothesized this result was due to the previously observed IBU cytotoxicity, which could have compromised the barrier integrity of the TM. This was experimentally confirmed by assessing tissue viability, TEER (a measure of barrier integrity), and histology. The data clearly show that IBU does indeed severely impact tissue viability and barrier integrity. This experiment also further supports the previous cytocompatibility assays and shows that I-HA does not damage TM or RWM models. This result also highlights the critical importance of covalent, chemical conjugation of IBU to HA. Although I-HA does not seem to readily cross the TM on its own, its use in conjunction with trans-tympanic delivery methods would warrant further investigation of I-HA as a topical ototherapeutic.

Overall, this study describes our approach to the generation of potential topical anti-inflammatory ototherapeutics. Our results highlight the selection process of I-HA, the conjugate that emerged as the most promising candidate for further investigation. Moreover, the study showed the importance of the chemical conjugation of IBU to HA, to the observed effects of I-HA. This approach yielded a compound with significantly reduced cytotoxicity, improved water solubility (vital for subsequent therapeutic formulation), and improved anti-inflammatory properties. In addition to the potential relevance of our findings for hearing research, we believe that the reduced cytotoxicity of I-HA may be of interest in other research fields. In particular, nonsteroidal anti-inflammatory drugs such as IBU are known to cause GI symptoms in up to 40% of users and 1%–2% of users experience serious complications (Sostres et al., 2013), making it one of the most common drug side-effects in the US. (Goldstein and Cryer, 2015) Therefore the conjugation to HA might constitute a strategy to mitigate such adverse effects, and potentially be used as an approach to formulate other therapeutics with reduced toxicity.

## Data Availability

The raw data supporting the conclusions of this article will be made available by the authors, without undue reservation.
