# Correction: Hyaluronic acid-ibuprofen conjugation: a novel ototherapeutic approach protecting inner ear cells from inflammation-mediated damage

**DOI:** 10.3389/fphar.2026.1924509

**Published:** 2026-09-21

**Authors:** Bhaskar Birru, Joachim G. S. Veit, Elizabeth M. Arrigali, Jack Van Tine, Emma Barrett-Catton, Zachary Tonnerre, Philippe Diaz, Monica A. Serban

**Affiliations:** 1 Department of Biomedical and Pharmaceutical Sciences, University of Montana, Missoula, MT, United States; 2 Montana Biotechnology Center (BIOTECH), University of Montana, Missoula, MT, United States

**Keywords:** inflammation, hyaluronic acid, ibuprofen, cochlea, ototherapeutic

The equation in **Materials and Methods**, section *2.7 Determination of HAC physicochemical properties* was erroneously given as $C_{\text{ii}}=\frac{C_{H}\left(\varepsilon_{H}-\varepsilon_{\text{ii}}\right)}{\left(\varepsilon_{\text{ii}}-\varepsilon_{i}\right)}$ .

The correct equation is $C_{\text{ii}}=\frac{C_{H}\left(\varepsilon_{H}-\varepsilon_{i}\right)}{\left(\varepsilon_{\text{ii}}-\varepsilon_{i}\right)}$ .

The original version of this article has been updated.

