# Supplementary material for: Hyaluronic acid-ibuprofen conjugation: a novel ototherapeutic approach protecting inner ear cells from inflammation-mediated damage
Source: Front Pharmacol. 2024 Feb 15;15:1355283. doi: 10.3389/fphar.2024.1355283 (PMC10902153; doi:10.3389/fphar.2024.1355283)
Supplement: Supplementary file 1 [file DataSheet1.PDF]

Supplementary Materials

Hyaluronic Acid-Ibuprofen Conjugation: A Novel Ototherapeutic Approach Protecting Inner Ear Cells from Inflammation-Mediated Damage

Bhaskar Birru[1]†, Joachim G.S. Veit[1,2]†, Elizabeth M. Arrigali[1]†, Jack Van Tine[1], Emma Barrett-Catton[1], Zachary Tonnerre[1], Philippe Diaz[1,2], *Monica A. Serban[1,2]

†These authors contributed equally to this work and share first authorship
[1]Department of Biomedical and Pharmaceutical Sciences, University of Montana, Missoula, MT
[2]Montana Biotechnology Center (BIOTECH), University of Montana, Missoula, MT

***Correspondence:**
Monica Serban
monica.serban@umontana.edu

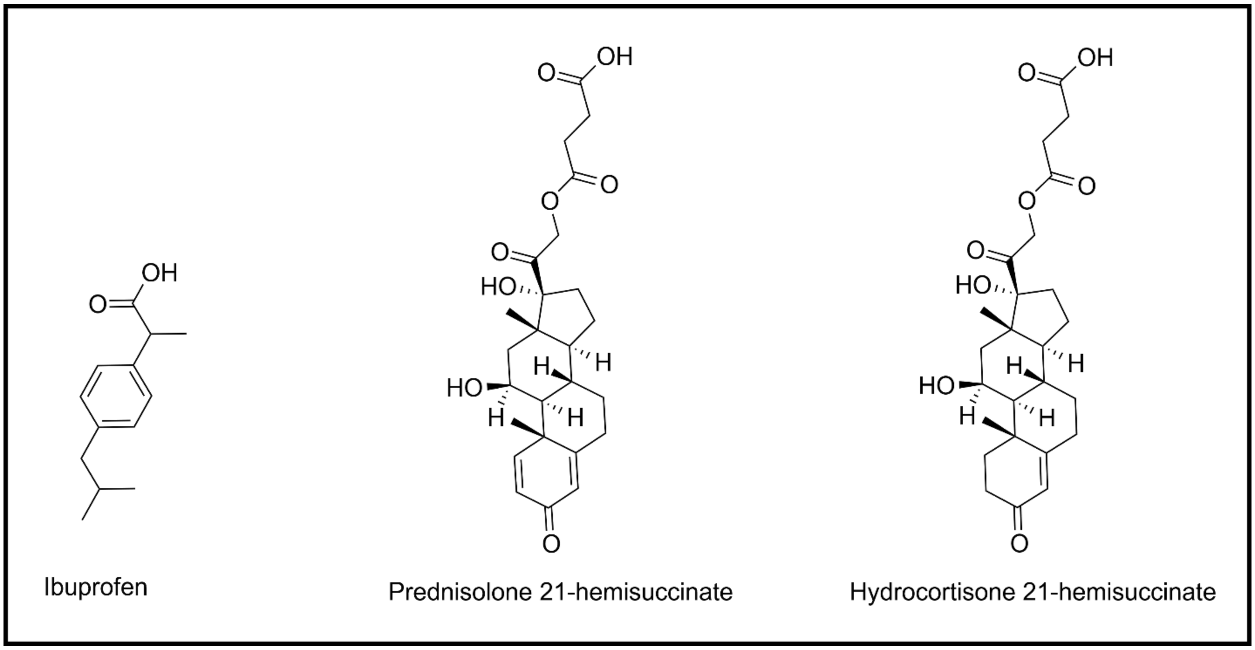

Supplementary Figure S1: Anti-inflammatory molecules utilized in this study.

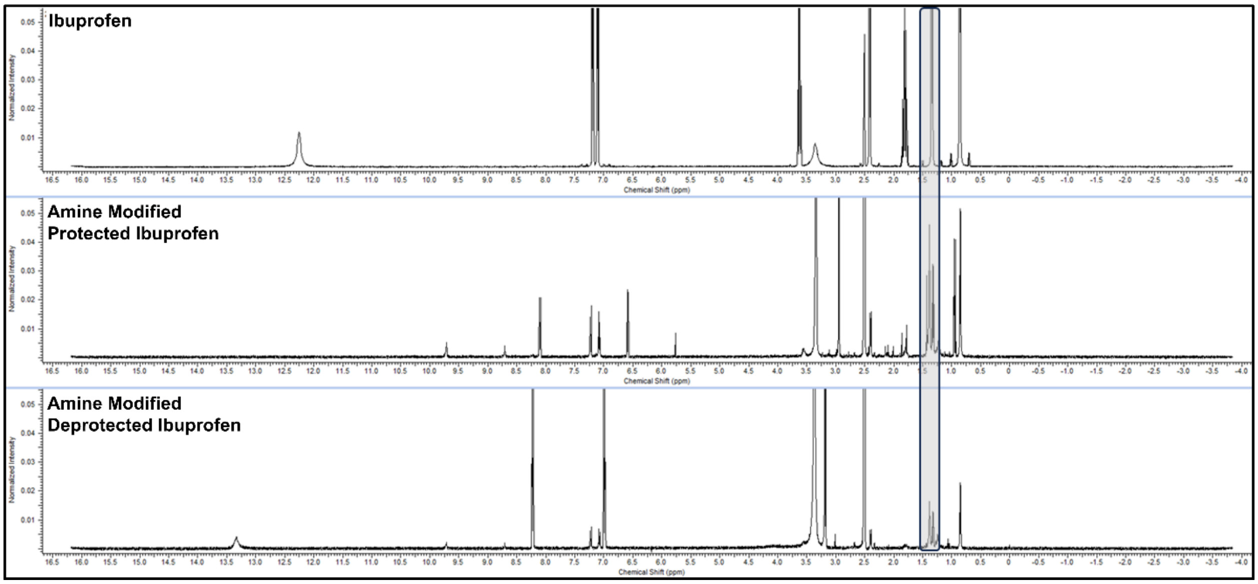

Supplementary Figure S2A: ^{1}H-NMR spectra for the amine modification of ibuprofen. All run in D6-DMSO. The top spectrum is the ibuprofen starting material. The middle spectrum is the amine modified ibuprofen with a BOC functionality protecting the amine. The bottom spectrum is the deprotected amine modified ibuprofen. The shaded box highlights the area of interest. Peaks at δ ~1.5 ppm originate from the BOC moiety. *BOC, tert-butyloxycarbonyl.*

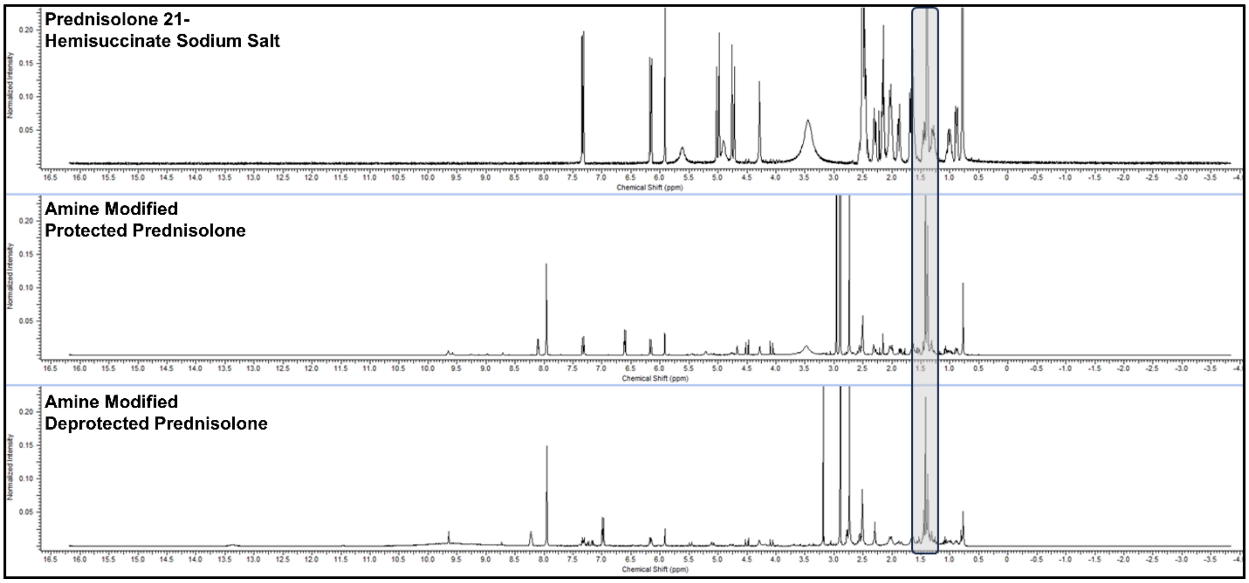

Supplementary Figure S2B: ^{1}H-NMR spectra for the amine modification of prednisolone 21-hemisuccinate sodium salt. All run in D6-DMSO. The top spectrum is the prednisolone starting material. The middle is the amine modified prednisolone with a BOC functionality protecting the amine. The bottom spectrum is after deprotection of the amine by removing the BOC protecting group. Shaded box highlights area of interest (δ~1.5 ppm) from protons originating from the BOC moiety. *BOC, tert-butyloxycarbonyl.*

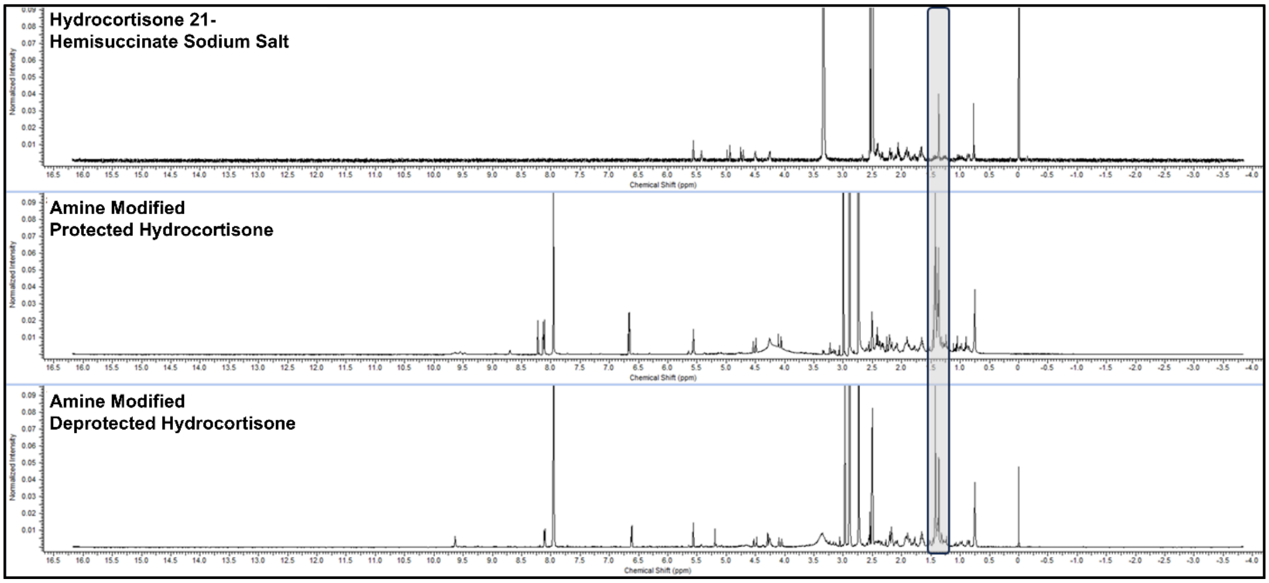

Supplementary Figure S2C: ^{1}H-NMR spectra for the amine modification of hydrocortisone 21-hemisuccinate sodium salt. All run in D6-DMSO. The top spectrum is the hydrocortisone starting material. The middle is the amine modified hydrocortisone with a BOC protecting the amine. The bottom is after removing the BOC functionality from the amine. Shaded box highlights area of interest (δ~1.5 ppm) from protons originating from the BOC moiety. *BOC, tert-butyloxycarbonyl.*

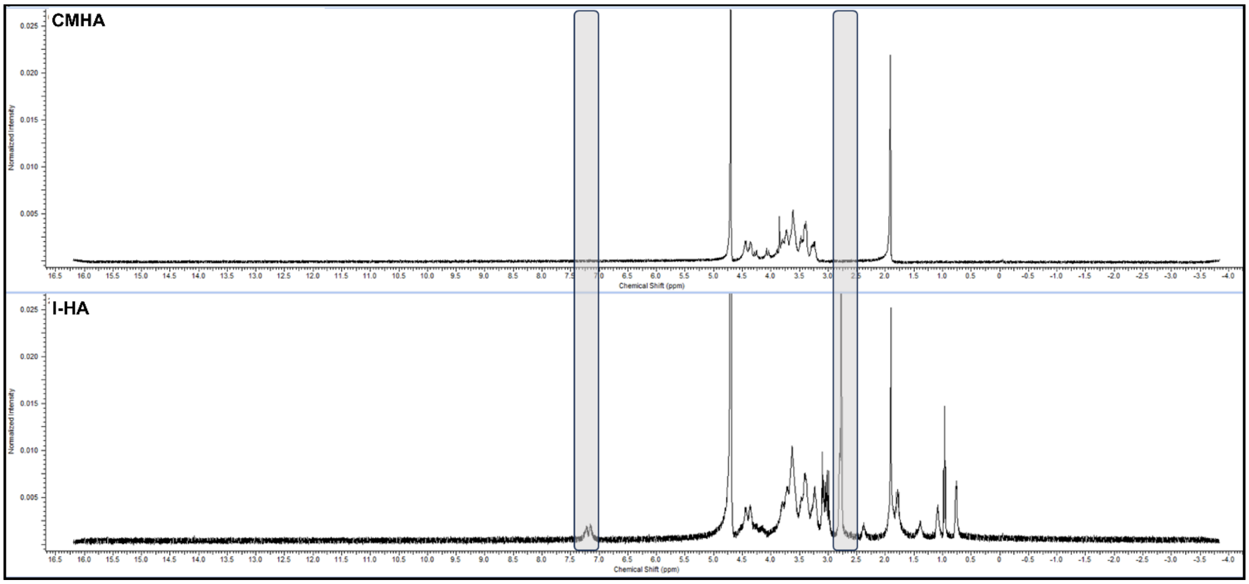

Supplementary Figure S3A: 1H-NMR spectra for the conjugation of the amine modified ibuprofen and CMHA to form I-HA. All run in deuterated water. The top is the CMHA starting material and the bottom spectrum is after the conjugation. The shaded boxes highlight the peaks of interest (~ 7.2 and 2.8 ppm) that are present only after amine modified ibuprofen has been conjugated to CMHA. *CMHA, carboxymethyl-hyaluronic acid; I-HA, Ibuprofen-CMHA conjugate.*

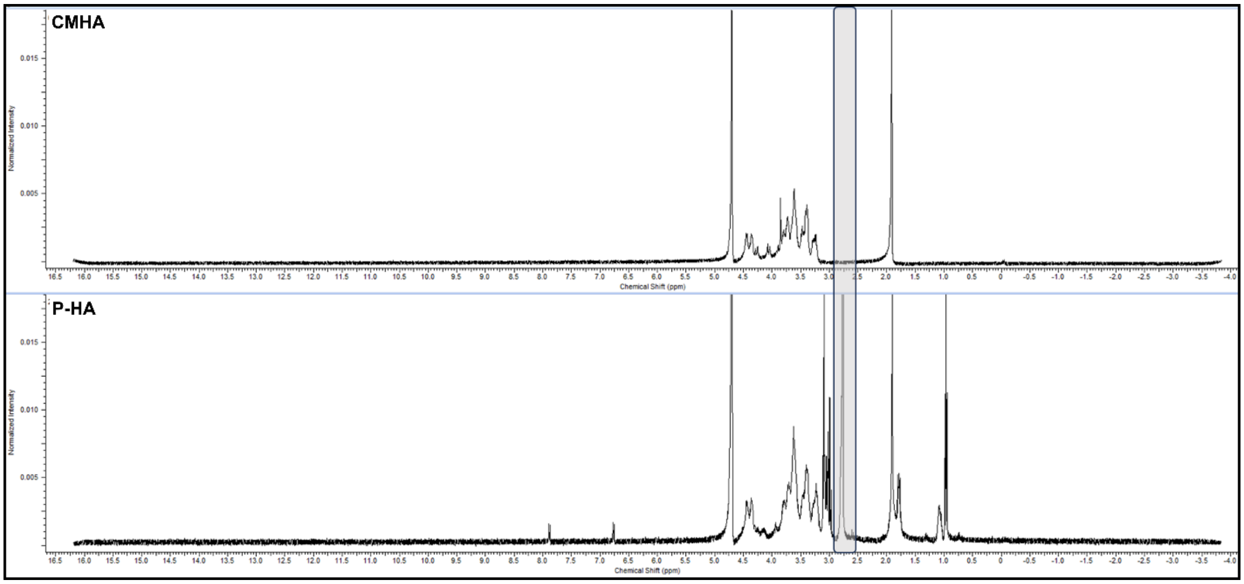

Supplementary Figure S3B: ^{1}H-NMR spectra for the conjugation of the amine modified prednisolone and CMHA to form P-HA. All run in deuterated water. The top is the CMHA starting material and the bottom spectrum is after the conjugation. The shaded box highlights the peak of interest (~ 2.8 ppm) that is present only after amine modified prednisolone has been conjugated to CMHA. *CMHA, carboxymethyl-hyaluronic acid; P-HA, prednisolone-CMHA conjugate.*

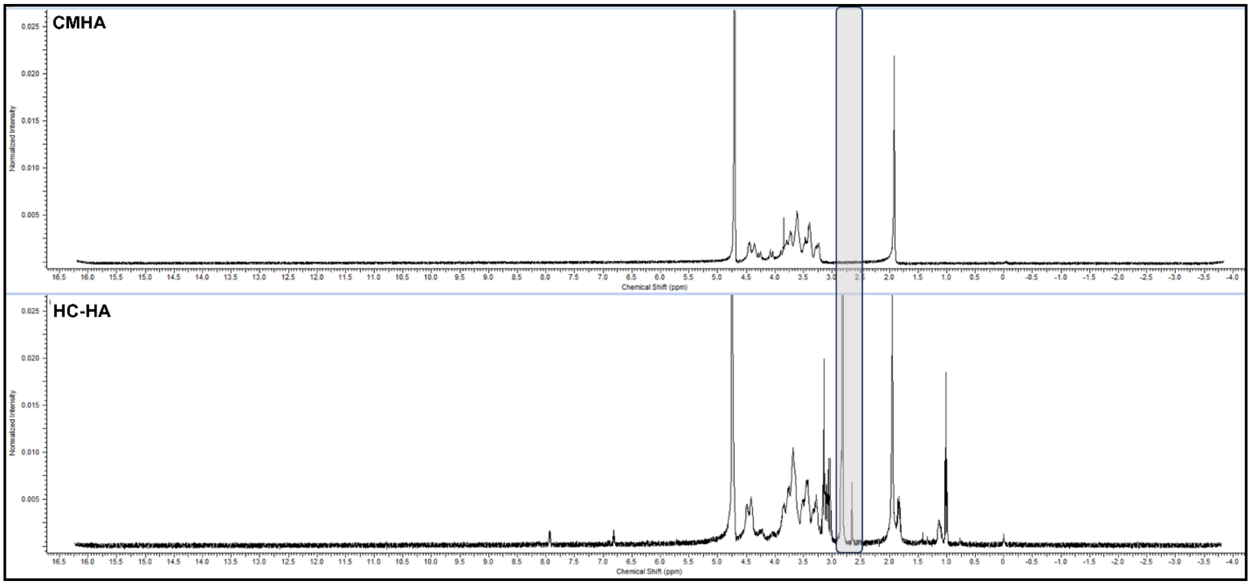

Supplementary Figure S3C: ^{1}H-NMR spectra for the conjugation of the amine modified hydrocortisone and CMHA to form HC-HA. All run in deuterated water. The top is the CMHA starting material and the bottom spectrum is after the conjugation. The shaded box highlights the peak of interest (~ 2.8 ppm) that is present only after amine modified hydrocortisone has been conjugated to CMHA. *CMHA, carboxymethyl-hyaluronic acid; HC-HA, hydrocortisone-CMHA conjugate.*

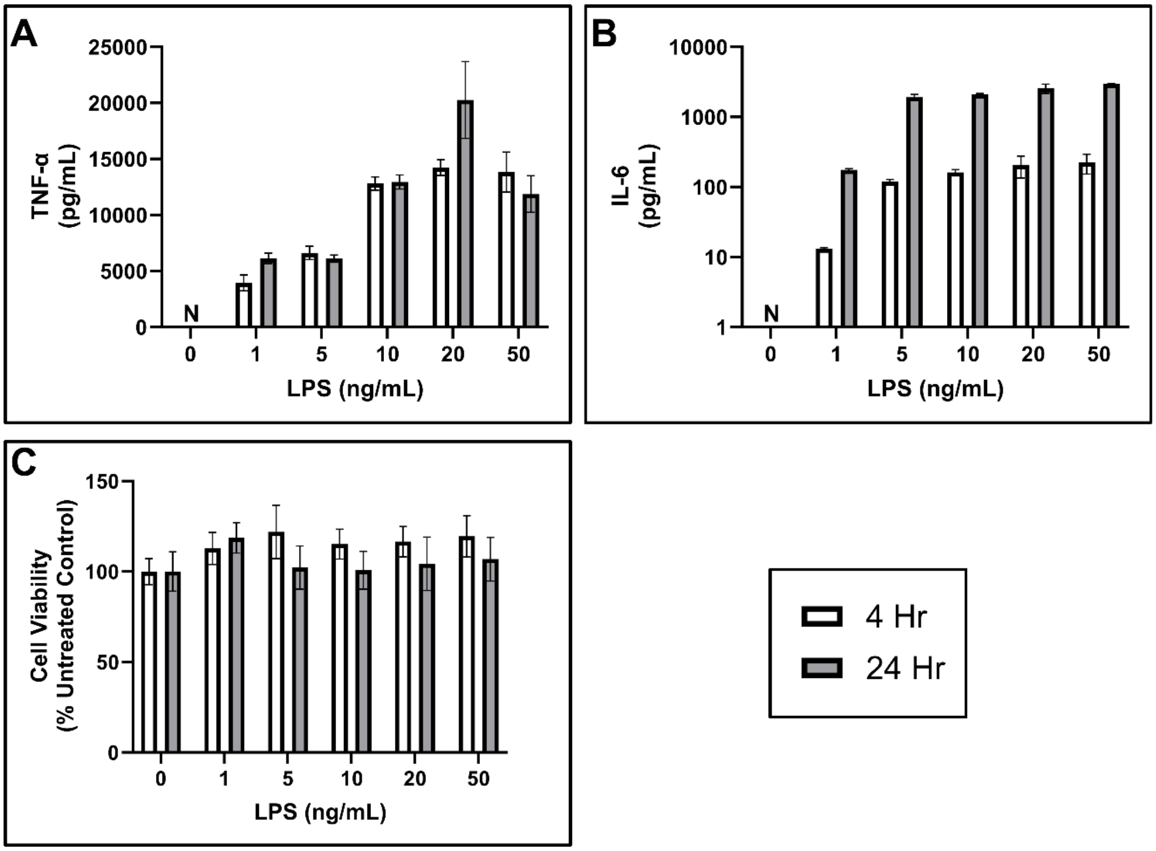

Supplementary Figure S4: LPS dose-response in macrophages. (**A**) IL-6 and (**B**) TNF-α release from RAW264.7 macrophages treated for 4 (white) or 24 (grey) hrs with various concentrations of LPS. n = 2 per group per dose. (**C**) Cell viability by MTS assay of macrophages treated for 4 (white) or 24 (grey) hrs with various concentrations of LPS. n = 4 per group per dose. All graphs show mean ± SD. *N, not detected; LPS, lipopolysaccharide.*

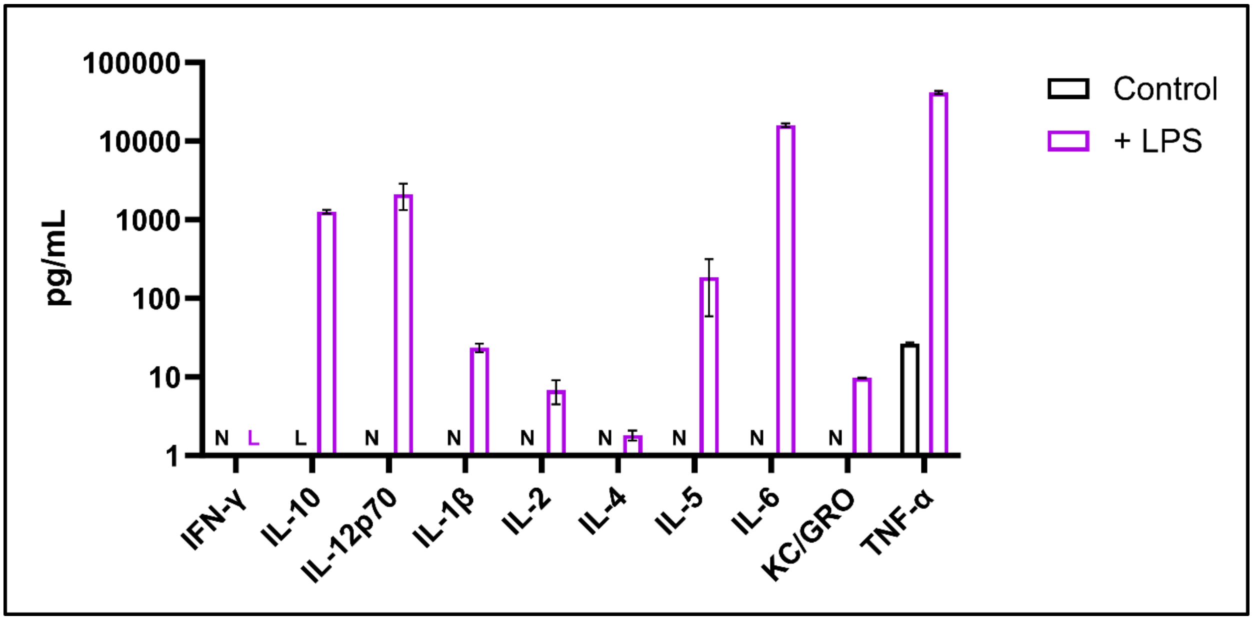

Supplementary Figure S5: Cytokine release in LPS-stressed macrophages. RAW264.7 macrophages were treated for 24 hrs with 10 ng/mL LPS, then the supernatant was analyzed for cytokine release in a panel of inflammatory cytokines. Mean ± SD; n = 2-3. *N, not detected; L, below lower quantification limit; LPS, lipopolysaccharide.*

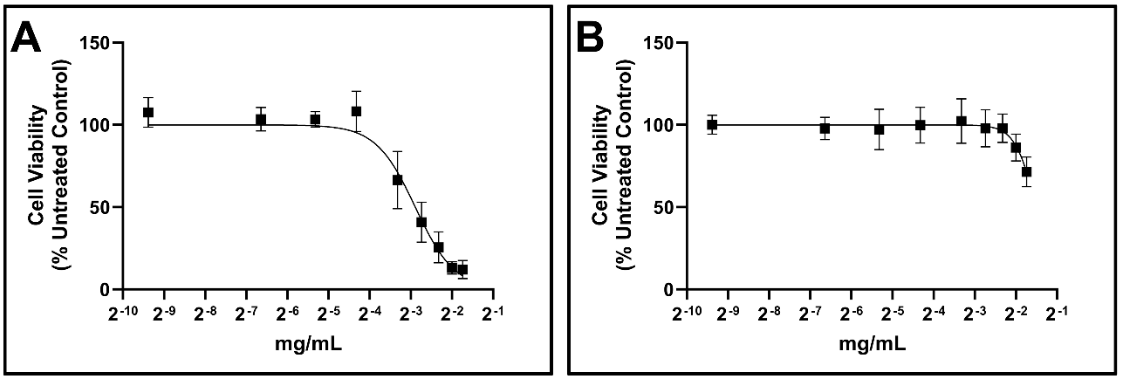

Supplementary Figure S6: Dose-response cell viability (MTS assay) curves of (**A**) RAW264.7 macrophages or (**B**) HEI-OC1 cells treated with IBU for 24 hrs. Maximum dose tested was at IBU's solubility limit in cell media (0.3 mg/mL). n = 7 - 8. Variable slope non-linear fit shown.

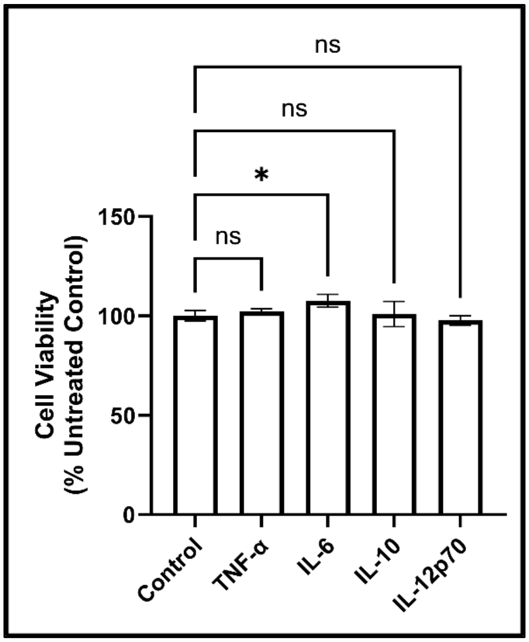

Supplementary Figure S7: Effect of individual cytokines from defined cytokine blend on cochlear cell viability. HEI-OC1 cells were treated with either TNF-α, IL-6, IL-10, or IL-12p70 at 42, 16, 1.3, and 2.1 ng/mL, respectively, for 24 hrs before measuring cell viability by MTS assay. Mean ± SD; n = 4; Brown-Forsythe and Welch one-way ANOVA test with Dunnett T3 Correction; *p<0.05, ns = not significant.

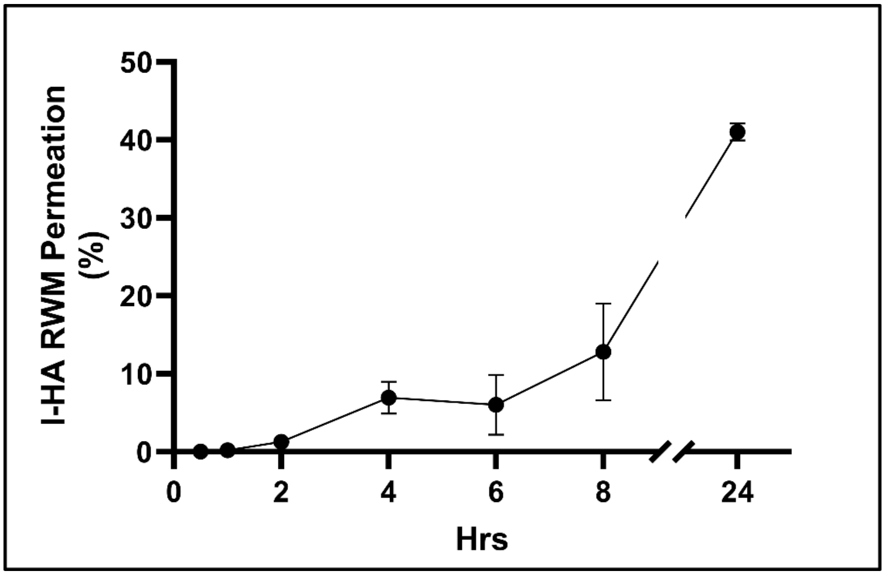

Supplementary Figure S8: Kinetics of I-HA permeation across in vitro RWM permeation model. n = 3 – 4.

Supplementary Table S1

M_w (kDa)	Conjugation Efficiency (% w/w)*	PDI (Đ)	dn/dc (mL/g)	ζ-potential (-mV)
28.3-29.3	12.0-12.9	1.312-1.445	0.1491-0.1499	25.85-27.69

*Values are shown as range of replicates. *% mass of IBU vs total I-HA mass. dn/dc, refractive index increment; M_w, weight-average molecular weight; PDI, polydispersity index.*
